# Mesothelioma-associated fibroblasts enhance proliferation and migration of pleural mesothelioma cells via c-Met/PI3K and WNT signaling but do not protect against cisplatin

**DOI:** 10.1186/s13046-022-02582-0

**Published:** 2023-01-23

**Authors:** Alexander Ries, Daniela Flehberger, Astrid Slany, Christine Pirker, Johanna C. Mader, Thomas Mohr, Karin Schelch, Katharina Sinn, Berta Mosleh, Mir Alireza Hoda, Balazs Dome, Helmut Dolznig, Georg Krupitza, Leonhard Müllauer, Christopher Gerner, Walter Berger, Michael Grusch

**Affiliations:** 1 Center for Cancer Research and Comprehensive Cancer Center, Medical University of Vienna, Borschkegasse 8a, 1090 Vienna, Austria; 2 Department of Analytical Chemistry, University of Vienna, Waehringer Straße 38, 1090 Vienna, Austria; 3 Joint Metabolome Facility, University of Vienna and Medical University of Vienna, Waehringer Guertel 38, 1090 Vienna, Austria; 4 ScienceConsult - DI Thomas Mohr KG, Enzianweg 10a, 2353 Guntramsdorf, Austria; 5 Department of Thoracic Surgery, Medical University of Vienna, Waehringer Guertel 18-20, Vienna, 1090 Austria; 6 National Korányi Institute of Pulmonology, Korányi Frigyes u. 1, Budapest, 1122 Hungary; 7 Department of Thoracic Surgery, National Institute of Oncology, Semmelweis University, Rath Gyorgy u. 7-9, Budapest, 1122 Hungary; 8 Institute of Medical Genetics, Medical University of Vienna, Waehringer Straße 10, 1090 Vienna, Austria; 9 Department of Clinical Pathology, Medical University of Vienna, Waehringer Guertel 18-20, 1090 Vienna, Austria

**Keywords:** Pleural mesothelioma, Cancer-associated fibroblasts, Co-cultures, Tumor microenvironment, WNT, PI3K, C-Met, HGF, Cisplatin, Pemetrexed

## Abstract

**Background:**

Pleural mesothelioma (PM) is an aggressive malignancy with poor prognosis*.* Unlike many other cancers, PM is mostly characterized by inactivation of tumor suppressor genes. Its highly malignant nature in absence of tumor driving oncogene mutations indicates an extrinsic supply of stimulating signals by cells of the tumor microenvironment (TME). Cancer-associated fibroblasts (CAFs) are an abundant cell type of the TME and have been shown to drive the progression of several malignancies. The aim of the current study was to isolate and characterize patient-derived mesothelioma-associated fibroblasts (Meso-CAFs), and evaluate their impact on PM cells.

**Methods:**

Meso-CAFs were isolated from surgical specimens of PM patients and analyzed by array comparative genomic hybridization, next generation sequencing, transcriptomics and proteomics. Human PM cell lines were retrovirally transduced with GFP. The impact of Meso-CAFs on tumor cell growth, migration, as well as the response to small molecule inhibitors, cisplatin and pemetrexed treatment was investigated in 2D and 3D co-culture models by videomicroscopy and automated image analysis.

**Results:**

Meso-CAFs show a normal diploid genotype without gene copy number aberrations typical for PM cells. They express CAF markers and lack PM marker expression. Their proteome and secretome profiles clearly differ from normal lung fibroblasts with particularly strong differences in actively secreted proteins. The presence of Meso-CAFs in co-culture resulted in significantly increased proliferation and migration of PM cells. A similar effect on PM cell growth and migration was induced by Meso-CAF-conditioned medium. Inhibition of c-Met with crizotinib, PI3K with LY-2940002 or WNT signaling with WNT-C59 significantly impaired the Meso-CAF-mediated growth stimulation of PM cells in co-culture at concentrations not affecting the PM cells alone. Meso-CAFs did not provide protection of PM cells against cisplatin but showed significant protection against the EGFR inhibitor erlotinib.

**Conclusions:**

Our study provides the first characterization of human patient-derived Meso-CAFs and demonstrates a strong impact of Meso-CAFs on PM cell growth and migration, two key characteristics of PM aggressiveness, indicating a major role of Meso-CAFs in driving PM progression. Moreover, we identify signaling pathways required for Meso-CAF-mediated growth stimulation. These data could be relevant for novel therapeutic strategies against PM.

**Supplementary Information:**

The online version contains supplementary material available at 10.1186/s13046-022-02582-0.

## Background

Pleural mesothelioma (PM) is an aggressive malignancy that arises from the mesothelial cells that line the pleural cavity [1]. These cells have a mesenchymal origin but grow in an epithelioid manner and express both mesenchymal and epithelial markers [2]. PM occurs in three main histological subtypes termed epithelioid, biphasic or sarcomatoid depending on the predominant display of more epithelioid versus more fibroblastoid cell shapes [3]. Inhalation of asbestos fibers is the most important risk factor for PM development [4]. Treatment options for PM are still very limited and the median overall survival of PM patients is little more than 1 year [5]. Around 10% of mesothelioma patients are carriers of a germline mutation of BRCA1 associated protein 1 (BAP1) and have a much better prognosis [6]. Treatment with cisplatin and pemetrexed has been the standard doublet chemotherapy for PM for many years but often shows limited efficacy [7]. Multimodality treatment protocols including chemotherapy, radiation, and surgery can be performed in selected patients with early stage disease. Recently, combination immunotherapy with ipilimumab and nivolumab was approved as a novel treatment option in PM with improved survival in a subgroup of patients [8].

Genetically, PM is characterized by inactivating mutations or deletions of tumor suppressor genes like BAP1, p16 or p53 [1]. Gain of function mutations in oncogenes like Ras or epidermal growth factor receptor (EGFR), in contrast, are rarely found in PM. On the one hand, the lack of oncogenic mutations makes it difficult to define suitable intervention points in tumor cells for targeted therapy approaches. On the other hand, it suggests that signals driving growth and migration of PM cells may be supplied by cells of the tumor microenvironment (TME). In many types of cancer, cancer-associated fibroblasts (CAF) are one of the most abundant cell types in the TME. They become activated by signals from the tumor cells and are believed to be derived from quiescent tissue resident fibroblasts, although other cellular sources such as bone marrow-derived mesenchymal stem cells, smooth muscle cells or adipocytes are also being discussed [9]. CAFs have been shown to regulate many aspects of tumor progression including proliferation, migration, invasion, and therapy response of tumor cells [10]. Little information is available so far about the characteristics of CAFs in PM and their impact on PM progression.

In the current study, we describe the isolation and the genetic and functional characterization of mesothelioma-associated fibroblasts (Meso-CAFs) from surgical specimens of PM patients. Meso-CAFs exhibit gene and/or protein expression patterns that distinguish them from normal lung fibroblasts and PM cells as well as from CAFs of lung, breast or colon cancer. Moreover, we demonstrate in 2D and 3D models that Meso-CAFs significantly enhance the growth and migration of PM cells in a pathway-dependent way but do not provide protection against cisplatin treatment.

## Methods

### Isolation of mesothelioma-associated fibroblasts

Tissue samples were collected from surgery specimens of PM patients who underwent surgical resection at the Department of Thoracic Surgery of the Medical University of Vienna. The study was approved by the ethics committee of the Medical University of Vienna (EK Nr. 904/2009) and written informed consent to use their tissue for research purposes has been obtained from all patients. To establish primary Meso-CAF cultures, the tumor tissue was minced into small pieces and incubated in cell culture flasks (T25) in RPMI-1640 growth medium (#R6504; Sigma-Aldrich, St. Louis, MO, USA) supplemented with 10% heat-inactivated fetal bovine serum (FBS) (Biowest, France) and antibiotics (100 U/ml penicillin and 100 μg/ml of streptomycin (1% P/S)) (Sigma-Aldrich, St. Louis, MO, USA). The tissue pieces were incubated in a humidified atmosphere (37 °C, 5% CO_2_) without agitation (for the first 7 days) to allow attachment of tumor pieces to the culture device and cell outgrowth. The phenotype of out-growing cells was validated by light microscopy. Flasks with fibroblastoid cells were selected for further cultivation and expansion. The cells were regularly washed with sterile phosphate-buffered saline (PBS, 1x) to remove cell debris.

### Cell culture

The fibroblastoid cells, which could be verified as Meso-CAFs (Meso109F, Meso125F, VMC59F) were all maintained in RPMI-1640 medium supplemented with 10% FBS within uncoated flasks. All primary Meso-CAFs used for experiments were at fewer than 15 passages. The human mesothelioma cell line MSTO-211H was purchased from the American Type Culture Collection (ATCC, Rockville, MD, USA), SPC212 was provided by Prof. R. Stahel (University of Zurich, Switzerland), and Meso84 as well as VMC23 were established at the Medical University of Vienna as recently described by Pirker et al. [11]. All PM cells were kept in RPMI-1640 medium supplemented with 10% FBS. Establishment of the primary cancer-associated fibroblasts CAF-3 from colorectal adenocarcinoma cultured in endothelial cell growth medium (EGM 2 MV; #CC-3202; Lonza, Basel, Switzerland) was previously described [12]. The human primary normal lung fibroblasts (NLFs) MRC-5 and Wi-38 were obtained from the ATCC, and cultivated in the respective growth medium according to the supplier’s protocol (MRC-5 in Dulbecco’s modified Eagle’s medium (DMEM) and Wi-38 in Eagle’s minimal essential medium (MEM) both supplemented with 10% FBS). All cells were incubated under normoxic cell culture conditions (37 °C, 5% CO_2_) in a humidified incubator, regularly passaged by trypsinization and routinely checked for *Mycoplasma* contamination. Human cell authentication was carried out for all cells using short tandem repeat DNA profiling analysis. A summary of all cell types used in the study is provided in Supplementary Table S1.

### Determination of doubling times

To evaluate the doubling times of Meso-CAFs, 7.5 × 10^4^ cells per well were seeded in a 12-well plate. Duplicates were trypsinized every 48 h and cell numbers were counted at different time points using a Neubauer cell counting chamber. Final doubling times of the cells were calculated from duplicated cell counts of at least four time points using the formula: doubling time [h] per time point = (time [h]) × log(2)/(log(cell count at time point)-log(seeded cell number)).

### Array-based comparative genomic hybridization

Microarray-based comparative genomic hybridization (array CGH) analysis of Meso-CAFs and CAF-3 was performed as described by Pirker et al. [11] and Mathieu et al. [13]. Genomic DNA was isolated from primary fibroblast cultures of about 80% cell confluence using QIAamp DNA Blood Mini Kit (Qiagen, Valencia, CA, USA). The genome analysis was carried out on 4x44K whole genome oligonucleotide-based microarrays (Agilent, Santa Clara, CA, USA) according to the manufacturer’s protocol, scanned on a G2505B Micro Array Scanner (Agilent, Santa Clara, CA, USA) and analyzed using the software Genomic Workbench (version 7.0) (Agilent, Santa Clara, CA, USA). Array CGH profiles of fibroblasts were checked for genomic alterations and compared to genome profiles of PM cell lines from a previous publication [11]. Array CGH data from Meso-CAFs and CAF-3 are available at ArrayExpress (https://www.ebi.ac.uk/biostudies/arrayexpress) under the accession number E-MTAB-12179.

### Quantitative real-time reverse transcription PCR

Commonly used CAF and PM markers were selected from the literature and their expression levels were quantified in Meso-CAFs, CAF-3 and NLFs (MRC-5, Wi-38), as well as in PM cells of different histological subtypes (VMC23, SPC212, MSTO-211H, Meso84) using quantitative real-time reverse transcription PCR (qRT-PCR). Total cellular mRNA was isolated from cell cultures of about 80% confluence with innuPREP RNA Mini Kit (Analytik Jena, Jena, Germany) and cDNA was synthesized with 2 μg of RNA using RevertAid RT Kit (Thermo Scientific, Thermo Fisher Scientific, Carlsbad, CA, USA), both according to the respective manufacturer’s instructions. The qRT-PCR was carried out with 1 μl of cDNA and appropriate primer pairs on a C1000 Touch Thermal Cycler (Bio-Rad Laboratories, Hercules, CA, USA) using SYBR Green PCR Master Mix (Applied Biosystems, Thermo Fisher Scientific, Carlsbad CA, USA). All primer sequences are listed in Supplementary Table S2. Glyceraldehyde-3-phosphate dehydrogenase (GAPDH) and β-Actin were used as reference genes for normalization and gene expression levels were calculated with the ΔCt method.

### Immunohistochemistry

Generation of paraffin-embedded cell blocks was done as previously described [14]. Sections were incubated with primary antibodies (BAP1, sc-28383 (Santa Cruz Biotechnology, Dallas, TX, USA), 1:200; calretinin, E7R60 (Cell Signaling Technology, Danvers, MA, USA), 1:50; CK8/18, C51 (Cell Signaling Technology, Danvers, MA, USA), 1:50; WT1, D817F (Cell Signaling Technology, Danvers, MA, USA), 1:100) at 4 °C overnight and antibody binding was detected with the UltraVison LP detection system (Lab Vison Corporation, Freemont, CA, USA).

### Next generation sequencing

DNA and RNA were isolated from the Meso-CAFs as described above for array CGH and qRT-PCR, respectively. Sequencing was performed with a 523 gene panel (TruSight Oncology 500 panel, Illumina Inc., San Diego, CA, USA) on an NextSeq 550 instrument (Illumina Inc). The list of investigated genes is shown as Supplementary Table S3. The sequences were analyzed with the Clinical Genomics Workspace bioinformatics pipeline from Pierian (Creve Coeur, MO, USA).

### Sanger sequencing

For Sanger sequencing, PCR amplicons spanning all loci containing variants in Meso109F were generated with Q5 proofreading polymerase (New England Biolabs, Ipswitch, MA, USA) using DNA from Meso109F cells and from whole blood of the same patient (isolated as described above for array CGH) as template. PCR products were purified and Sanger sequencing was done by Microsynth Austria GmbH (Vienna, Austria). PCR primers are listed in Supplementary Table S2 and were also used as sequencing primers.

### Whole genome gene expression microarrays

Total RNA of Meso-CAFs and CAF-3 was extracted from primary cell cultures of about 80% confluence with RNeasy Mini Kit (Qiagen Sciences, Germantown, MD, USA) according to the respective protocol of the manufacturer. Whole-genome gene expression analysis was carried out as described previously by Pirker et al. [11] and Mathieu et al. [13] using 4x44K whole genome oligonucleotide-based gene expression microarrays (Agilent, Santa Clara, CA, USA) and a G2505B Micro Array Scanner (Agilent, Santa Clara, CA, USA). Gene expression data of Meso-CAFs was compared to CAF-3 and to data of 31 PM cell lines available from a previous publication [11]. Analysis and comparison of expression data was conducted in R [15], and differentially expressed genes between cell types (Meso-CAF, CAF-3, PM) were determined using “limma” package [16] as previously described by Mohr et al. [17]. Genes with multiple oligonucleotide probes on the array were summarized to the probe with maximal interquartile range using the package “genefilter” [18]. Additional plots were created using GraphPad Prism 8.0 (GraphPad Software, San Diego, CA, USA). Whole genome gene expression array data from Meso-CAFs and CAF-3 are available at ArrayExpress (https://www.ebi.ac.uk/biostudies/arrayexpress) under the accession number E-MTAB-12177.

### Mass spectrometry-based proteomics

Three different protein fractions (supernatant, cytoplasmic and nuclear proteins) of Meso-CAFs (Meso109F, Meso125F, VMC59F) and NLFs (MRC-5, Wi-38) were analyzed in three biological replicates. The cells were cultivated in T25/T75 flasks until exhibiting 80–90% of confluence and the growth medium was exchanged to the respective FBS-free medium 6 hours prior to protein extraction. Protein fractioning was conducted as described by Slany et al. [19]. The supernatant containing the secreted proteins was initially collected, and the cells were lysed using isotonic lysis buffer supplemented with protease inhibitors and mechanical shear stress application with a 23 g syringe. The nuclei were separated from the cytoplasmic protein fraction by centrifugation and all protein fractions were individually precipitated in ethanol at − 20 °C overnight. After centrifugation, protein pellets were dissolved in sample buffer (7.5 M urea, 1.5 M thiourea, 4% CHAPS, 0.05% SDS, 100 mM dithiothreitol) and concentrations of distinct protein fractions were assessed using Bradford assay (Bio-Rad Laboratories, Hercules, CA, USA). Enzymatic in-solution digestion of proteins into peptides with Trypsin/Lys-C (Promega Corporation, Fitchburg, WI, USA) and sample clean-up on SDB-RPS StageTips was performed based on the protocol from Humphrey et al. [20] with minor changes. Peptide samples were subjected to LC-MS/MS analyses on a Dionex Ultimate 3000 nano LC-system (Thermo Scientific, Thermo Fisher Scientific, Carlsbad, CA, USA) coupled to a timsTOF pro mass spectrometer (Bruker Daltonics, Bruker Corporation, Billerica, MA, USA). All samples were run as technical replicates and data analysis for protein identification was performed using MaxQuant 1.6.17.0 [21] employing the Andromeda software searching against the UniProt Database for human proteins (version 12/2019 with 20,380 entries). The mass spectrometry proteomics data were submitted to the ProteomeXchange Consortium via the PRIDE partner repository and can be accessed with the dataset identifier PXD035987, PXD036017 and PXD036127 [22]. Statistical data analysis and the generation of figures was conducted in R with the package “DEP” [23] and by the use of the software Perseus (version 1.6.14.0) [24, 25]. Additional plots were created using GraphPad Prism 8.0.

The proteins of the supernatant, cytoplasmic and nuclear fraction were additionally filtered for actively secreted, membrane-associated and DNA-binding proteins, respectively, by comparing the protein data sets with published human data from protein databases (UniProt, Human Protein Atlas). Moreover, the secretome data of lung-CAFs [26], colon-CAFs [27] and breast-CAFs [28] was collected from the literature, filtered for actively secreted proteins as described above and the annotated genes of the identified proteins in the secretomes of different CAFs and Meso-CAFs were compared using Venny 2.1 [29]. To measure the similarity between the secretomes, Jaccard similarity coefficients between different gene sets were calculated. Gene ontology (GO) enrichment analysis was performed using DAVID functional annotation tool [30, 31] and biological processes associated with differentially expressed proteins of Meso-CAFs, NLFs and CAFs from other tumor entities were determined.

A more detailed methodologic description of the proteome analysis is available as online supplement (Supplementary Methods).

### Transduction of tumor cells with green fluorescent protein

The cDNA of green fluorescent protein (GFP) was integrated into the genome of PM cells using retroviral transduction to introduce stable expression of the fluorescence protein. Generation of retrovirus particles in HEK293 cells and transduction of target cells was performed as previously described [32]. Briefly, HEK293 cells, which were maintained in DMEM supplemented with 10% FBS were transfected by calcium phosphate co-precipitation to take up the vector pQCXIP (Clontech, Mountain View, CA, USA) containing the GFP sequence as well as a puromycin resistance gene and the two helper plasmids pVSV-G (Clontech, Mountain View, CA, USA) and p-gag-pol-gpt [33]. Supernatants of the transfected HEK293 cells containing the retroviral particles were used to transduce SPC212 and MSTO-211H cells in the presence of polybrene (8 μg/ml), followed by a selection of GFP-positive cells with puromycin treatment (0.8 μg/ml).

### Generation of 2D co-cultures

GFP-tagged tumor cells were either seeded alone or together with Meso-CAFs in a ratio of 1:100 (96-well plate: 150 tumor cells, 1.5 × 10^4^ Meso-CAFs; 48-well plate: 300 tumor cells, 3 × 10^4^ Meso-CAFs). Bright field and fluorescence images of the wells were taken every 24 h for 3 days, either manually using a Nikon Eclipse Ti2 microscope with a DS-Fi3 camera (Nikon, Tokyo, Japan) or automatically using the IncuCyte S3 Live-cell Analysis System (Sartorius, Göttingen, Germany). Definiens Developer XD Software (Definiens, Carlsbad, CA, USA) was used to quantify the number of tumor cells on the images via automated cell counting based on cell size and GFP signal. Tumor cell growth over time was evaluated by determining the cell number percentage at different time points compared to 0 h.

### Generation of 3D co-cultures

3D cell cultures were generated as described by Dolznig et al. [12]. GFP-tagged tumor cells were either embedded alone or together with Meso-CAFs in a matrix of collagen in a ratio of 1:100 (3 × 10^3^ tumor cells, 3 × 10^5^ Meso-CAFs). The collagen solution was prepared on ice by mixing 2 mg/ml collagen type I (rat tail; Corning, Bedford, MA, USA), 10x PBS and 0.05% methylcellulose, as well as NaOH (1 M) to neutralize the pH (7.2–7.4). The cells were gently resuspended in the solution and the cell suspension was transferred into a silicone gel casting mold inside a 6 cm plastic dish. A nylon mesh insert was centrally placed in the middle of each culture to prevent collagen gel contraction by the fibroblasts. After incubating the cultures for 1 h at 37 °C (5% CO_2_), the collagen gels were polymerized and the casting molds were removed. The remaining collagen gel cylinders with the cells were maintained in RPMI-1640 medium with 10% FBS under regular culture conditions and the growth medium was exchanged every second day. Bright field and fluorescence images of the cultures were taken every 24 h for 6 days using a Nikon Eclipse Ti2 microscope with a DS-Fi3 camera. The pictures were taken at five different non-overlapping positions of the cultures and several layers per location to reflect the whole 3D culture. Quantification of tumor cells and determination of cell growth over time was performed as described above.

### Analysis of cell migration

2D cultures were generated as described above and GFP^+^ tumor cells were monitored by videomicroscopy using the IncuCyte S3. Images of the wells were taken every 30 minutes for a total duration of 60 h. Movements of single GFP^+^ tumor cells were manually tracked using ImageJ to obtain coordinates for each individual cell and time point. The DiPer migration tool for Microsoft Excel was used to analyze the migratory behavior of tumor cells in presence or absence of Meso-CAFs including migrated distance, mean squared displacement (MSD), directionality ratio (DR), and plots of origin [34].

### Analysis of effects mediated by conditioned medium

Conditioned medium (CM) was prepared by cultivating Meso-CAFs or NLFs under conventional culture conditions and collecting the supernatant. When the cells exhibited a confluence of about 80%, they were incubated in fresh RPMI-1640 medium supplemented with 10% FBS for 72 h. The collected supernatant was centrifuged for 10 minutes at 1500×g to remove cell debris and either immediately used for experiments or stored at − 80 °C for later use. GFP^+^ tumor cells were seeded in 48-well plates (300 tumor cells per well) and treated for 30 h with a mix of CM and fresh RPMI-1640 medium supplemented with 10% FBS in a ratio of 1:1 (500 μl of each per well). Fresh growth medium was added to exclude effects from depleted growth medium constituents in the CM alone and ensure full nutrition of the tumor cells over the experiment. Fresh growth medium alone was used as control and all experiments were performed in triplicates. The generation of 2D cultures as well as the evaluation of cell growth and migration was carried out as described above using IncuCyte S3 and Definiens or ImageJ. Images were taken every 5 h or every 30 minutes for the analysis of growth or migration, respectively.

### Analysis of inhibitor treatment effects

2D cultures in 48-well plates were treated with various small molecule inhibitors of signaling pathways and were imaged every 24 h for 72 h using IncuCyte S3. Tumor cell growth over time was determined as described above and effects of distinct signaling inhibition in presence or absence of Meso-CAFs were evaluated. Final working concentration of each inhibitor was determined by a stepwise increase of concentrations until a reduction of tumor cell growth of at least 20% could be observed either in the cultures with or in those without Meso-CAFs (assessed by automated image analysis) without leading to a visually assessed decrease in the cell confluence of the Meso-CAFs. The following inhibitors were used at indicated final concentrations: TGFβ receptor type I inhibitor galunisertib (Selleckchem, Houston, TX, USA, 10 μM), the EGFR inhibitor erlotinib (Selleckchem, 1 μM), the PI3K inhibitor LY-294002 (Selleckchem, 5 μM), the c-Met inhibitor crizotinib (MedChem Express, Monmouth Junction, NJ, USA, 1 μM (SPC212) / 2 μM (MSTO-211H)), the MEK inhibitor U0126 (Selleckchem, 0.1 μM (SPC212) / 0.25 μM (MSTO-211H)), the FAK inhibitor BI 853520 (Boehringer Ingelheim, Ingelheim, Germany, 5 μM (SPC212) / 2 μM (MSTO-211H)), the NF-κB inhibitor BAY 11–7082 (Biomol, Hamburg, Germany, 5 μM (SPC212) / 2 μM (MSTO-211H)), the FGF receptor inhibitor erdafitinib (MedChem Express, 0.5 μM), the triple angiokinase inhibitor nintedanib (MedChem Express, 0.5 μM (SPC212) / 2 μM (MSTO-211H)), the MMP inhibitor ilomastat/GM6001 (MedChem Express, 1 μM (SPC212) / 10 μM (MSTO-211H)), the TEAD inhibitor K-975 (MedChem Express, 1 μM (SPC212) / 3 μM (MSTO-211H)), and the PORCN inhibitor WNT-C59 (MedChem Express, 5 μM). DMSO was used as vehicle control in the experiments.

### Analysis of cisplatin and pemetrexed treatment effects

2D and 3D cultures were treated with the chemotherapeutic alkylating agent cisplatin (Sigma-Aldrich, St. Louis, MO, USA) and cultures were imaged at 0 h, 48 h and 72 h. Three different concentrations were used in the 2D approach (1 μM, 3 μM, 10 μM), whereas the lowest concentration was omitted in the 3D models due to limited effects. Additionally, pemetrexed (MedChem Express) was used in 2D cultures at four different concentrations (0.3 μM, 1 μM, 3 μM, 10 μM). Effects of cisplatin and pemetrexed on tumor cell growth were evaluated as described above.

### Statistical analysis

All cell behavior experiments were performed in at least three independent replicates and are shown as means and SEM, unless stated otherwise. Statistical analyses were conducted using GraphPad Prism 8.0. Differences were evaluated by ANOVA and were considered statistically significant at a *p*-value < 0.05.

## Results

### Cell populations with a fibroblast morphology isolated from PM specimens show normal genotypes and CAF marker expression

**Fig. 1:**
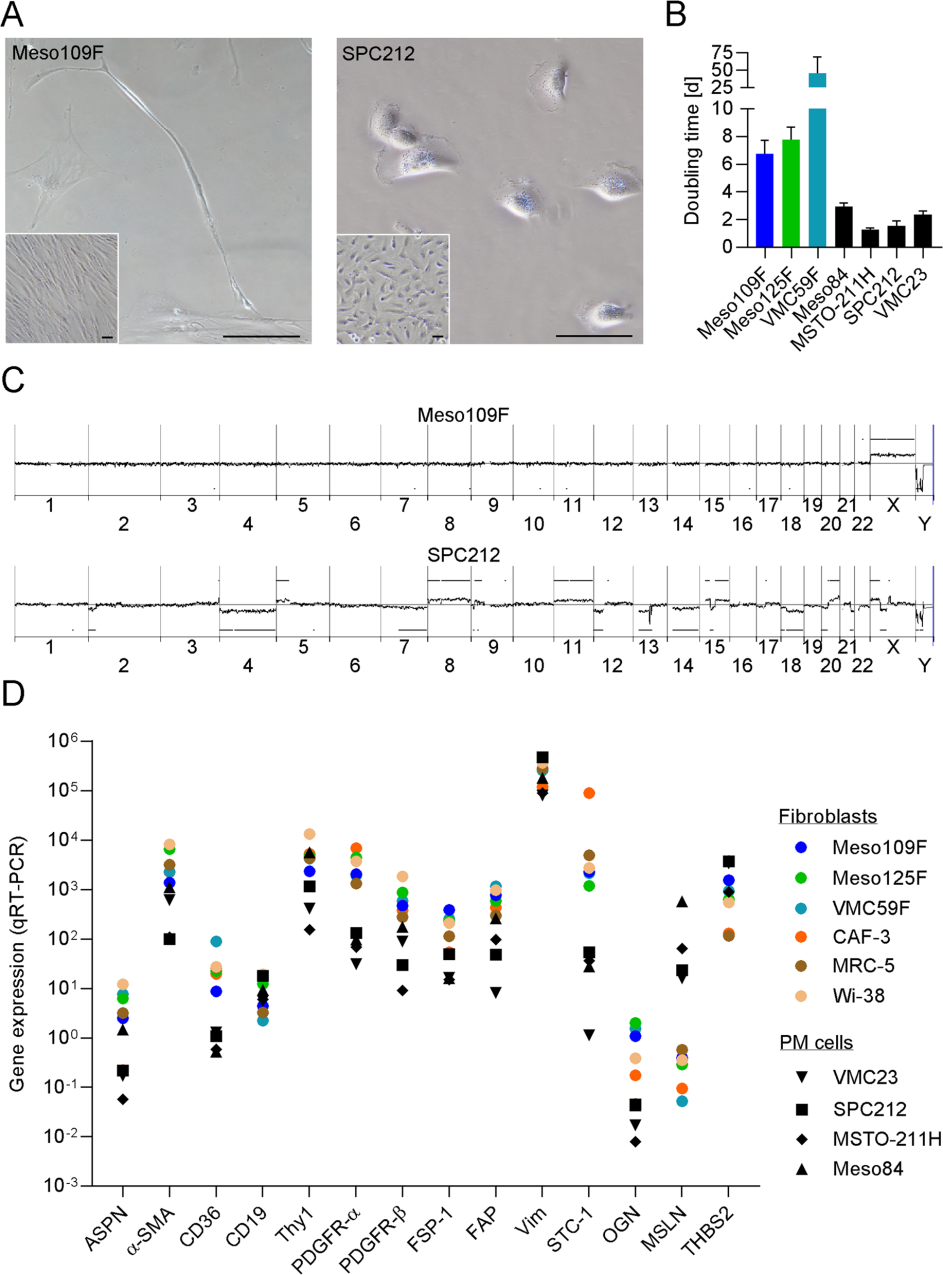
Cell populations with a fibroblastoid morphology isolated from PM patients lack chromosomal abnormalities and express markers of cancer-associated fibroblasts. **A** Micrographs of cultured Meso109F and the biphasic PM cell line SPC212 were taken at low and high (insets) density. Scale bar = 100 μm. **B** Doubling times (mean and SEM) for the Meso-CAFs, the sarcomatoid PM cell line Meso84, the biphasic PM cell lines SPC212 and MSTO-211H, and the epithelioid PM cell line VMC23 were calculated as described in Materials and Methods. **C** Array comparative genomic hybridization data for Meso109F and SPC212. Gains and losses of gene dose across all chromosomes compared to the reference DNA are depicted as elevations and depressions of the horizontal line, respectively. **D** The indicated cell lines were subjected to qRT-PCR to determine the expression of genes previously described as fibroblast or PM marker genes. Each dot represents the mean of at least three biological replicates. Gene expression data were normalized to GAPDH and β-Actin expression and are shown as 2^–ΔCt^ * 10^5^

Tumor pieces from PM patients were mechanically minced and transferred to cell culture flasks. Cell populations outgrowing from three of the samples showed a fibroblast-like morphology and were thus designated Meso109F, Meso125F, and VMC59F. They appeared morphologically different from epithelioid, biphasic and sarcomatoid PM cells (Fig. 1A, Supplementary Fig. S1). They showed doubling times of 6.7, 7.5, and 45.4 days for Meso109F, Meso125F, and VMC59F, respectively (Fig. 1B).

To determine whether the isolated fibroblast-like cells have a normal diploid genotype lacking the typical genomic aberrations associated with PM, we performed array CGH experiments. The results showed no chromosomal abnormalities in Meso109F (Fig. 1C), Meso125F, and VMC59F (Supplementary Fig. S2), as well as in a bona fide colon CAF (CAF-3 [12]), which was used as control. In contrast, all PM cell lines including SPC212 showed a high number of chromosomal gains and losses/deletions as expected (Fig. 1C, Supplementary Fig. S2). Next, we determined the expression of several marker genes previously described in the literature to be associated with either CAFs or PM cells. For comparison, we included CAF-3, the normal lung fibroblasts (NLFs) MRC-5 and Wi-38, as well as the PM cell lines VMC23, SPC212, MSTO-211H and Meso84 in this analysis. Meso109F, Meso125F and VMC59F, as well as the other fibroblasts (CAF-3, MRC-5, Wi-38) expressed high levels of the fibroblast markers alpha-smooth muscle actin (α-SMA, ACTA2), CD36, fibroblast activation protein alpha (FAP), platelet-derived growth factor receptor (PDGFR)-α and -β and stanniocalcin (STC-1) but very low levels of the PM marker mesothelin (MSLN) (Fig. 1D). Of the investigated markers, CD36, PDGFR-α, STC-1 and MSLN showed a good overall separation between fibroblasts and PM cells, whereas other mesenchymal markers like FAP or vimentin (VIM) were highly expressed also in PM cells (Fig. 1D).

Immunohistochemical stainings of formalin-fixed and paraffin-embedded cell pellets for markers used in routine diagnostic pathology [35] demonstrated the absence of the mesothelioma markers cytokeratin (CK) 8/18, calretinin and Wilms tumor (WT) 1 and nuclear staining of BAP1, indicative of its wild-type status, in all three Meso-CAFs (Supplementary Fig. S3). The sarcomatoid PM cell line Meso84, in contrast, expressed both CK8/18 and WT1.

To check for genetic variants, including germline variants found in around 10% of PM patients [6], we analyzed 532 genes by next generation sequencing in Meso109F, Meso125F and VMC59F. Meso125F and VMC59 did not harbor any pathogenic or likely pathogenic DNA sequence variants. In Meso109F, a FANCL (Fanconi Anemia Complementation Group L) DNA sequence variant of uncertain clinical significance was recognized (c.1096_1099dupATTA; p.T367Nfs; variant allele frequency (VAF) 40.7%). This variant is seen in up to 0.5% of the general population according to the Genome Aggregation Database (gnomAd). The variant is located at the C-terminal end of the gene. Additionally, variants of uncertain significance were detected in EP300 (E1A Binding Protein P300) (c.5347_5375del29; p.K1783Cfs*90; VAF 31.4%), MSH3 (MutS Homolog 3) (c.2319-1G > A; p.?; VAF 46.8%) and NTRK3 (Neurotrophic Receptor Tyrosine Kinase 3) (c.863A > G; p.N288S; VAF 34.5%). Gene fusions were absent in all three Meso-CAFs. By subsequent Sanger sequencing, the Meso109F variants in FANCL and MSH3, but not those in EP300 and NTRK3 were seen also in the blood of the corresponding patient, identifying the former as germline variants (Supplementary Fig. S4).

In addition, Meso109F was tested for tumorigenicity in immunocompromised mice and, as expected, did not form tumors. Together, these data clearly indicate that the isolated cell populations represent fibroblasts associated with mesothelioma and we hence termed them Meso-CAFs.

### Meso-CAFs show gene expression patterns distinct from PM cells and colon CAFs

**Fig. 2:**
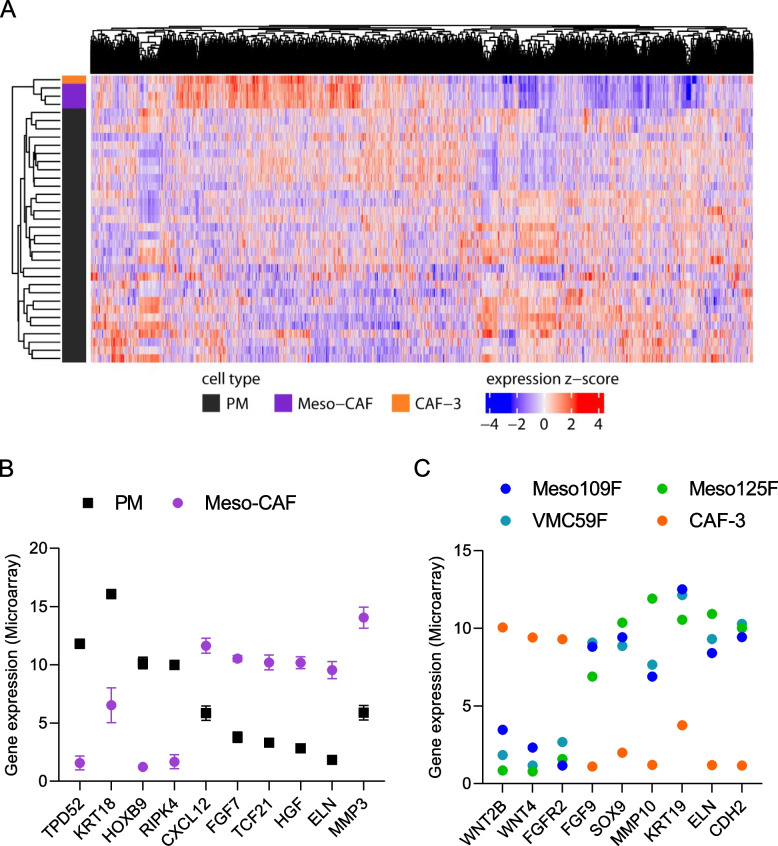
Gene expression profiles of Meso-CAFs differ from PM cells and colon CAFs. **A** Unsupervised clustering of 31 PM cell lines, the three Meso-CAFs (Meso109F, Meso125F, VMC59F) and the colon cancer-associated fibroblasts CAF-3. **B** Selected genes showing either significantly lower or higher expression in Meso-CAFs compared to PM cells. Values represent means and SEM of log_2_ transformed hybridization signals for each category. **C** Selected genes showing either depressed or elevated expression in all three Meso-CAFs compared to CAF-3. Values represent log_2_ transformed hybridization signals

As a next step to compare Meso-CAFs to PM cells and other CAFs and screen for potential molecular interactions between Meso-CAFs and PM cells, we performed whole genome gene expression microarrays of Meso109F, Meso125F, VMC59F and CAF-3 and compared them to the transcriptomes of a panel of previously characterized PM cell lines [11]. Unsupervised clustering of Meso-CAFs, CAF-3, and 31 PM cell lines showed that the three Meso-CAFs clustered closely together and were clearly separated not only from the PM cluster but also from CAF-3 (Fig. 2A). The same result was also obtained when not the entire transcriptomes but a list of known CAF/PM markers was used for clustering (Supplementary Fig. S5).

Gene expression analysis identified 77 genes that showed differential expression between Meso-CAFs and PM cells when a fold change > 100 and a statistical threshold of *p* < 0.05 was applied and 94 genes that showed > 100-fold change in gene expression level between Meso-CAFs and CAF-3 (Supplementary Tables S4 and S5). Genes highly expressed across PM cells but low in Meso-CAFs included the tumor protein D52 (TPD52) and its interaction partner T-cell differentiation protein 2 (MAL2), the keratins 18 and 8, the homeobox transcription factor B9 (HOXB9) and receptor interacting serine/threonine kinase 4 (RIPK4) (Fig. 2B, Supplementary Table S4). Genes highly expressed in Meso-CAFs compared to PM included the intermediate filament elastin (ELN), the transcription factor 21 (TCF21) and a number of genes coding for secreted proteins like the chemokine CXCL12, the matrix metalloproteinase (MMP) 3, and the growth factors fibroblast growth factor (FGF) 7 and hepatocyte growth factor (HGF) (Fig. 2B). Most of these factors still provided a very good separation between Meso-CAFs and PM cells when only the three sarcomatoid PM cell lines of our PM panel were considered (Supplementary Fig. S6). When comparing Meso-CAFs with CAF-3, the WNT pathway genes WNT2B and WNT4 and the FGF receptor 2 were high in CAF-3 but low in all three Meso-CAFs, whereas FGF9, keratin 19, ELN and N-cadherin were highly expressed in all Meso-CAFs but low in CAF-3 (Fig. 2C, Supplementary Table S5).

### Meso-CAFs show differences in proteomes and secretomes compared to normal lung fibroblasts and CAFs from colon, breast and lung cancer

**Fig. 3:**
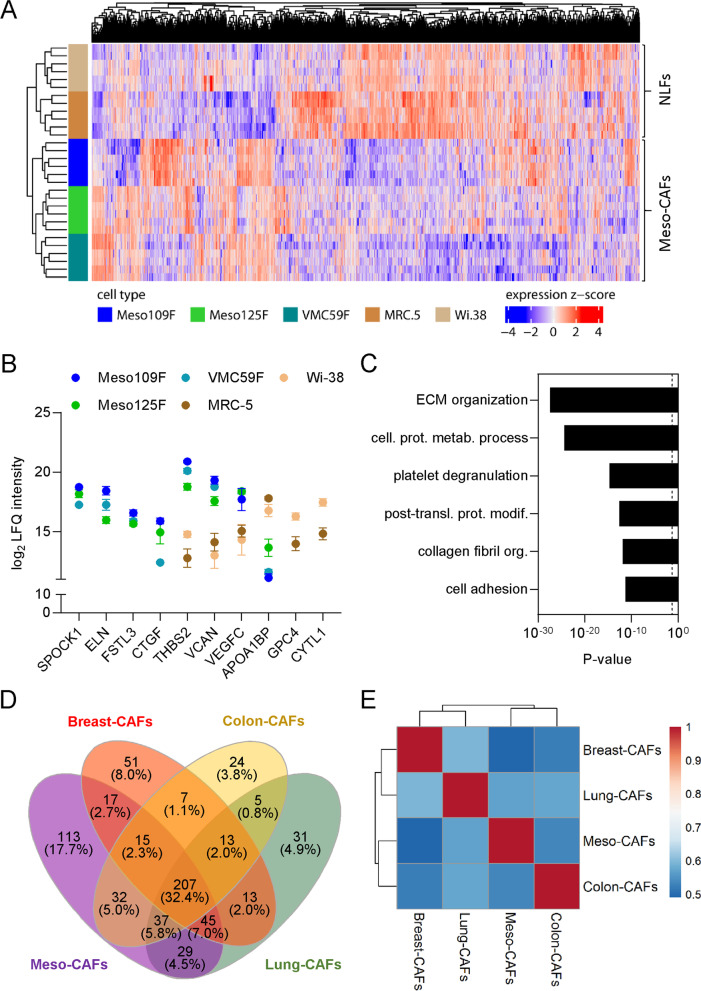
Secretome analysis reveals differences and overlaps with normal lung fibroblasts and CAFs from colon, lung and breast cancer. **A** Proteins in the supernatant fractions of Meso-CAFs and normal lung fibroblasts (NLF) were subjected to unsupervised clustering. **B** Selected secreted proteins with differential expression between Meso-CAFs and NLFs. Values are shown as mean and SEM of 3 biological replicates performed in duplicates. **C** Proteins only identified in Meso-CAFs or significantly upregulated in Meso-CAFs compared to NLFs were subjected to Gene Ontology (GO) analysis and the 6 most significantly associated biological processes are shown. **D** Venn diagram showing the overlaps in secreted proteins identified in CAFs from the indicated cancer types. **E** Heatmap of Jaccard similarity coefficients indicating the degree of secretome overlap between CAFs from the indicated cancer types

Next, we characterized the proteome of Meso109F, Meso125F, VMC59F, and, for comparison, the NLFs MRC-5 and WI-38. We performed mass spectrometry analysis and identified 5018, 4453 and 1931 proteins in the nucleus, cytoplasm and supernatant of Meso-CAFs, respectively, and comparable numbers of proteins in the respective fractions of NLFs (Supplementary Tables S6, S7 and S8). Volcano plots showed a high number of significantly up- and downregulated proteins between Meso-CAFs and NLFs in the supernatants and the cytoplasmic fractions but only a much smaller number in the nuclear fractions (Supplementary Fig. S7). Unsupervised cluster analysis of the supernatant protein fractions grouped together the three Meso-CAFs on the one hand and the two NLFs on the other (Fig. 3A). To focus on the most relevant proteins for cell type differentiation or potential interactions with tumor cells, proteins in the supernatant were filtered for secreted proteins according to Uniprot and Protein Atlas annotation, proteins in the cytoplasm were filtered for membrane proteins, and proteins in the nuclear fraction were filtered for DNA-binding proteins. This resulted in 227 DNA-binding proteins identified in the nuclear fraction, 1833 membrane proteins in the cytoplasmic fraction and 515 secreted proteins in the supernatant of Meso-CAFs (Table 1). Of the 515 secreted proteins identified in Meso-CAFs, 94 were unique to Meso-CAFs (not found in NLFs), 67 were significantly upregulated and 68 downregulated in Meso-CAFs compared to NLFs. Forty-six secreted proteins were only found in NLFs but not in Meso-CAFs. The respective numbers for membrane proteins and DNA-binding proteins are shown in Table 1. Secreted proteins identified in Meso-CAFs but not NLFs or significantly upregulated in Meso-CAFs compared to NLFs included several matricellular proteins like testican-1 (SPOCK1) or connective tissue growth factor (CTGF) as well as the lymphangiogenesis inducer vascular endothelial growth factor C (VEGFC) (Fig. 3B, Supplementary Table S9). When we performed a GO term analysis with all proteins upregulated in or exclusive to Meso-CAFs versus NLFs, extracellular matrix (ECM) organization and cell adhesion were among the most significantly associated biological process categories (Fig. 3C). GO term analysis results of secreted proteins downregulated in Meso-CAFs compared to NLFs or found only in NLFs and of differentially expressed membrane proteins and DNA-binding proteins are shown in Supplementary Fig. S8.

**Table 1:** Proteins identified in Meso-CAFs versus normal lung fibroblasts (NLFs)

	Found only in NLFs	Found only in Meso-CAFs	Found in Meso-CAFs and NLFs	Upregulated in Meso-CAFs (*p* < 0.05)	Downregulated in Meso-CAFs (*p* < 0.05)
Nucleus	187 (3.6%)	702 (13.5%)	4316 (82.9%)	6	20
DNA binding	4	59	168	0	0
Cytoplasm	142 (3.1%)	290 (6.3%)	4163 (90.6%)	269	258
Membrane	53	112	1721	135	103
Supernatant	582 (23.2%)	245 (9.7%)	1686 (67.1%)	111	585
Secreted	46	94	421	67	68

Since PM is a cancer of mesodermal origin, we hypothesized that Meso-CAFs might show protein expression patterns different from fibroblasts associated with epithelial cancers such as lung cancer, colon cancer or breast cancer. We focused on secreted proteins and retrieved secretome data for lung-CAFs, colon-CAFs and breast-CAFs from the literature [26–28]. We applied the same filters for secreted proteins as for our Meso-CAF data and determined the overlaps in identified secreted proteins. Two hundred and seven proteins were common to all CAF types, whereas 113 proteins were unique to Meso-CAFs (Fig. 3D). To visualize similarity between different CAF types, Jaccard similarity coefficients were calculated and plotted as heatmap. This placed Meso-CAFs closer to colon and lung-CAFs than to breast-CAFs but did not indicate a greater overall separation between Meso-CAFs and CAFs from epithelial cancers than between CAFs from different epithelial cancer types (Fig. 3E). “Cell adhesion” and “ECM organization” as well as “chondrocyte differentiation” and “ECM organization” were the two most significantly associated pathways for proteins common to all CAF types and proteins exclusive to Meso-CAFs, respectively (Supplementary Fig. S9). Presence of selected proteins in the secretome of Meso-CAFs and other CAF types is shown in Supplementary Fig. S10.

### Meso-CAFs increase growth and migration of PM cells

**Fig. 4:**
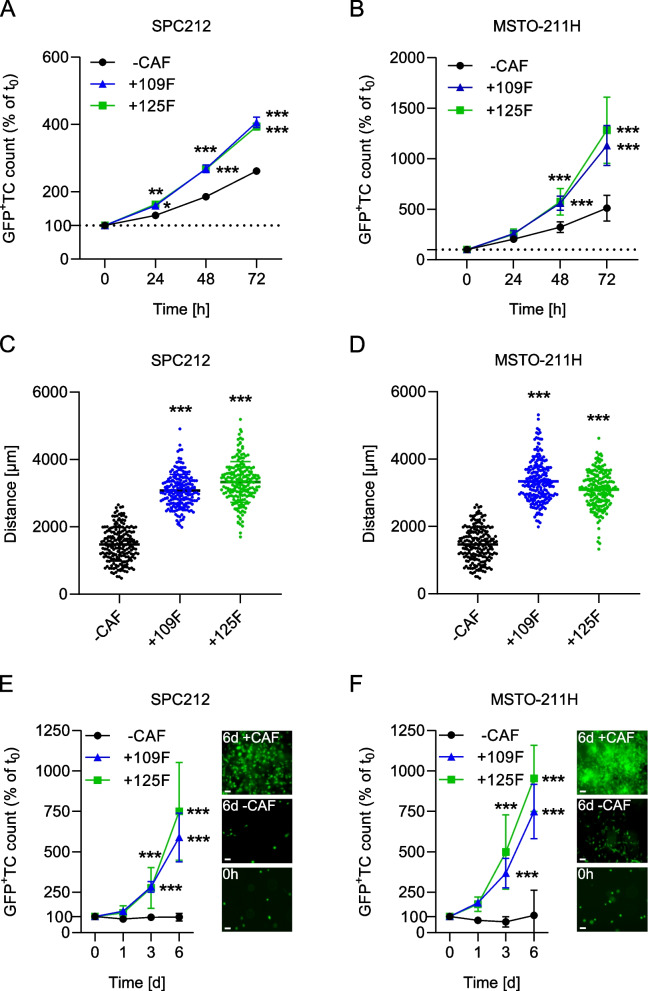
Meso-CAFs enhance the growth and migration of PM cells. Green fluorescent protein tagged (GFP^+^) SPC212 cells (**A**) and MSTO-211H (**B**) were cultured in the absence of CAFs (−CAF) or in the presence of Meso109F (+109F) or Meso125F (+125F). Micrographs were taken every 24 h and numbers of GFP^+^ tumor cells (TC) were determined by automated image analysis. GFP^+^ SPC212 (**C**) and GFP^+^ MSTO-211H (**D**) were cultured in the absence of CAFs (−CAF) or in the presence of Meso109F (+109F) or Meso125F (+125F). Individual tumor cells (*n* > 190 per condition) from three biological replicates were manually tracked for 60 h. Each dot represents the migrated distance of one individual cell. GFP^+^ SPC212 (**E**) and GFP^+^ MSTO-211H (**F**) were cultured in 3D collagen gels in the absence of CAFs (−CAF) or in the presence of Meso109F (+109F) or Meso125F (+125F). Micrographs were taken after 1, 3 and 6 days and numbers of GFP^+^ TC were determined by automated image analysis. Representative images of GFP^+^ TC in 3D collagen gels at 0 h (bottom) and after 6 days either in absence of Meso-CAFs (−CAF) (middle) or in the presence of Meso125F (+CAF) (top). Scale bar = 250 μm. *** *p* < 0.001, TC number in the presence of Meso-CAFs versus TC number in the absence of Meso-CAFs, one-way ANOVA with Dunnett’s multiple comparisons test (**C, D**) or two-way ANOVA with Tukey’s multiple comparisons test (**A, B, E, F**)

To assess the impact of co-culturing PM cell lines with Meso-CAFs, we initially used 2D monolayer cultures. We observed a significant growth stimulation of the PM cells when either SPC212 or MSTO-211H were co-cultured with Meso109F or Meso125F (Fig. 4A and B). The degree of stimulation was comparable in the two PM cell lines with both Meso109F and Meso125F. Subsequently, we assessed the influence of Meso-CAFs on PM cell migration and found that co-culture with Meso109F or Meso125F significantly increased the migrated distance of both PM cell lines (Fig. 4C and D). There were also significant differences in the mean square displacement but not in the straightness of cell trajectories in both PM cell lines upon co-culture with Meso-CAFs (Supplementary Fig. S11).

In order to evaluate whether stimulation of tumor cell expansion is also seen under more physiological culture conditions, we used a model where PM cells and fibroblasts were cultivated in a collagen 3D matrix. Here, both PM cell lines showed almost no growth when cultured in the absence of Meso-CAFs but robust growth when cultured together with either Meso109F or Meso125F (Fig. 4E and F). Due to their slow proliferation, only a limited number of experiments could be performed with VMC59F cells, but those that could be performed showed a growth-stimulating effect on MSTO-211H cells that was comparable to the other two Meso-CAF cultures in the 2D and the 3D model (Supplementary Fig. S12).

### Inhibition of c-Met/PI3K or WNT signaling decreases Meso-CAF-mediated growth stimulation of PM cells

**Fig. 5:**
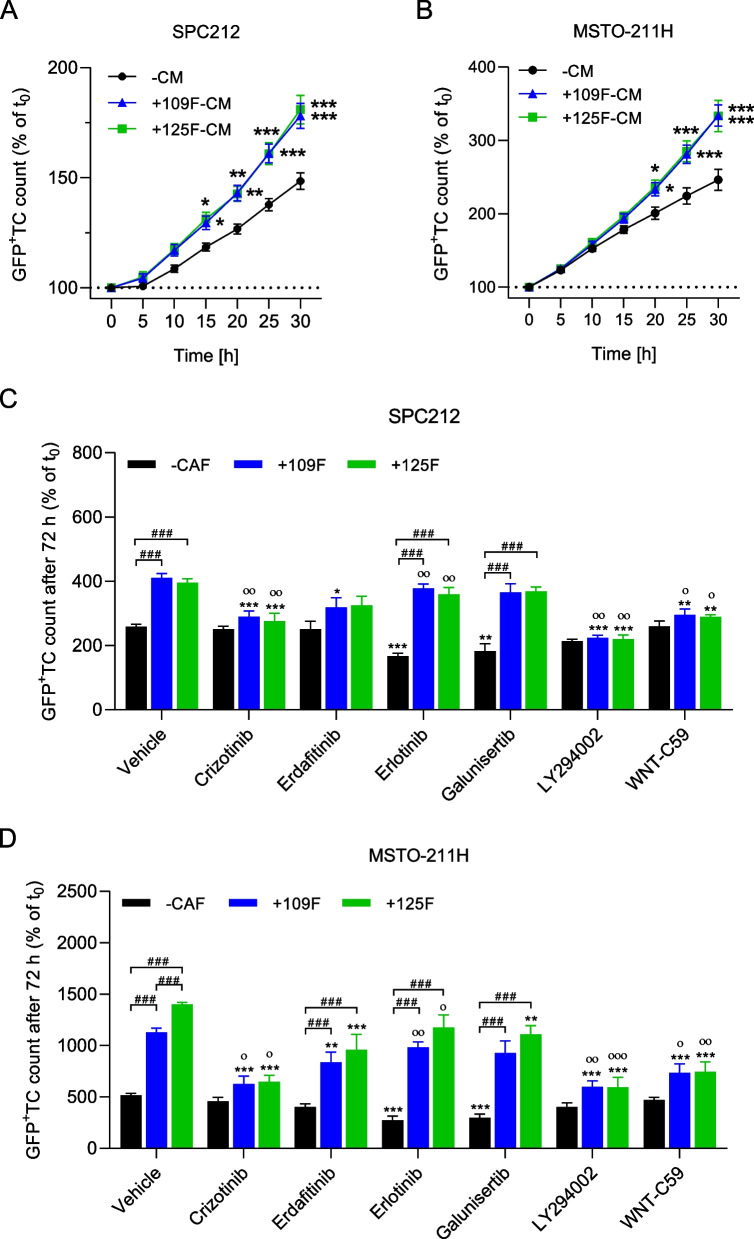
Crizotinib, LY294002 and WNT-C59 block the growth stimulation of PM cells by Meso-CAFs. Green fluorescent protein tagged (GFP^+^) SPC212 cells (**A**) and MSTO-211H (**B**) were incubated without conditioned medium (−CM) or with CM conditioned by Meso109F (+109F-CM) or Meso125F (+125F-CM). Micrographs were taken every 5 h and numbers of GFP^+^ tumor cells (TC) were determined by automated image analysis. * *p* < 0.05, ** *p* < 0.01, *** *p* < 0.001 TC number in the presence versus absence of CM, two-way ANOVA with Tukey’s multiple comparisons test. GFP^+^ SPC212 (**C**) or MSTO-211H (**D**) were cultured as monolayers in the absence of CAFs (−CAF) or in the presence of Meso109F (+109F) or Meso125F (+125F) and treated with crizotinib, erdafitinib, erlotinib, galunisertib, LY294002, WNT-C59 or vehicle (DMSO). Numbers of GFP^+^ TC after 72 h were calculated by automated image analysis. ** *p* < 0.01, *** *p* < 0.001 inhibitor treated versus vehicle treated, ^#^*p* < 0.05, ^##^*p* < 0.01, ^###^*p* < 0.001 growth in presence of Meso-CAFs versus growth in absence of Meso-CAFs, ° *p* < 0.05, °° *p* < 0.01, °°° *p* < 0.001 percent inhibition in presence of Meso-CAFs versus percent inhibition in absence of Meso-CAFs, one-way ANOVA with Tukey’s multiple comparisons test

Since Meso-CAFs stimulated growth and migration of PM cells in co-culture and produced multiple secreted signaling molecules, we aimed to elucidate the contributing mechanisms. As a first step, we explored whether Meso-CAF-CM and co-culturing had similar growth stimulating effects on PM cells. CM of Meso109F and Meso125F significantly stimulated the growth of SPC212 or MSTO-211H over a period of 30 h, indicating that, indeed, signaling molecules secreted by Meso-CAFs are instrumental in stimulating PM cell growth (Fig. 5A and B). Since NLFs also secrete signaling molecules, we determined the impact of their CM on PM cells and compared it to the impact of Meso-CAF-CM. NLF-CM showed stimulation of PM cell growth when compared to growth medium alone, which, however, only reached statistical significance for Wi-38 in SPC212 and MRC-5 in MSTO-211H cells (Supplementary Fig. S13). Nevertheless, Meso-CAF-CM showed a significantly stronger growth stimulation when compared to CM of both NLFs in MSTO-211H and to CM of MRC-5 in SPC212. Similarly, CM of Meso-CAFs and NLFs significantly stimulated cell migration of SPC212 and MSTO-211H, but, again, the effects of Meso-CAF-CM were significantly stronger compared to those of NLF-CM (Supplementary Fig. S13), suggesting that secretome differences between NLFs and Meso-CAFs are relevant for their growth- and migration-stimulating activity.

Next, we hypothesized that blockade of specific pathways might abrogate the growth promoting effect of Meso-CAFs on PM cells. Hence, we selected a panel of inhibitors targeting pathways previously associated with PM aggressiveness and used them to treat PM cells in the presence and absence of Meso-CAFs for 72 h. At non-toxic concentrations for the Meso-CAFs (Supplementary Fig. S14) the inhibitors still showed an effect on PM cells either in the absence or presence of Meso-CAFs. Of the 12 inhibitors tested, the c-Met inhibitor crizotinib, the phosphoinositide 3-kinase (PI3K) inhibitor LY294002 and the WNT signaling inhibitor WNT-C59 significantly reduced the growth stimulating effect of Meso-CAFs on PM cells in co-culture, but did not significantly affect PM cells alone (Fig. 5C and D). A similar trend (but no significant effect) to inhibit Meso-CAF-mediated growth stimulation of PM cells was observed with the FGF receptor inhibitor erdafitinib. In contrast, the EGFR inhibitor erlotinib had a significant effect on PM cells alone but not on PM cells co-cultured with Meso-CAFs, and a similar trend (but no significant effect) was seen for the transforming growth factor beta (TGF-β) receptor inhibitor galunisertib (Fig. 5C and D). The multikinase inhibitor nintedanib, the MEK inhibitor U0126, the focal adhesion kinase (FAK) inhibitor BI853520, the MMP inhibitor ilomastat, the nuclear factor k-light-chain-enhancer of activated B cells (NF-κB) inhibitor BAY11–7082 and the hippo pathway inhibitor K-975 impaired the growth of PM cells to various degrees, but no differences dependent on the absence versus presence of Meso-CAFs were observed (Supplementary Fig. S15). Full time course of data of the inhibitor treatment experiments are shown in Supplementary Figs. S16 and S17.

### Meso-CAFs do not protect PM cells against cisplatin treatment

**Fig. 6:**
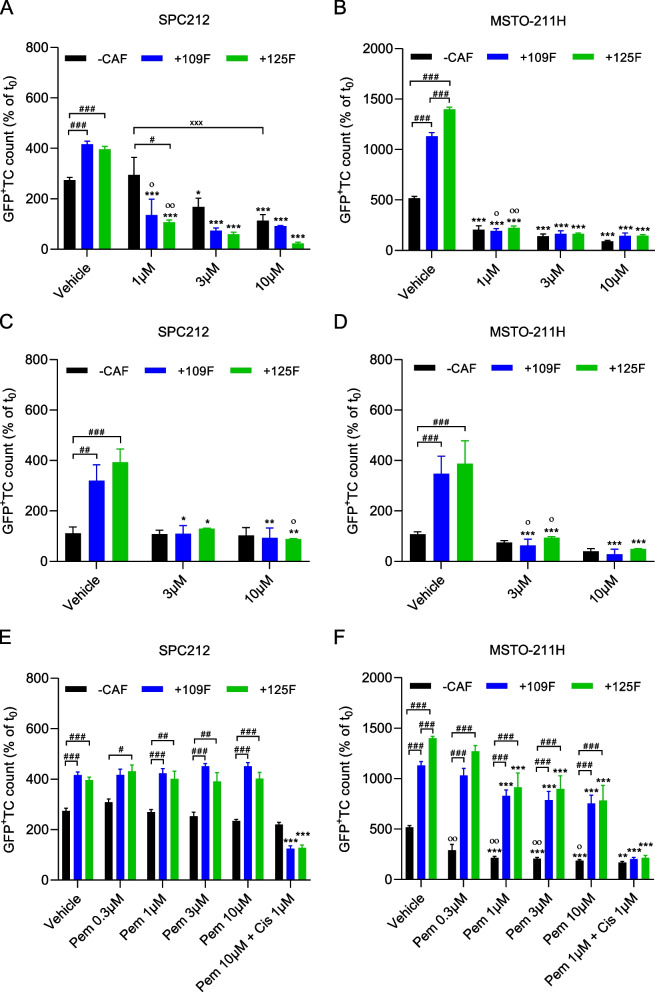
Meso-CAFs do not protect PM cells against cisplatin. GFP^+^ SPC212 (**A**) or GFP^+^ MSTO-211H (**B**) were cultured as monolayers in the absence of CAFs (−CAF) or in the presence of Meso109F (+109F) or Meso125F (+125F) and treated with 1, 3 or 10 μM cisplatin or vehicle (DMSO). Micrographs were taken after 72 h and numbers of GFP^+^ tumor cells (TC) were determined by automated image analysis. GFP^+^ SPC212 (**C**) and GFP^+^ MSTO-211H (**D**) were cultured in 3D collagen gels in the absence of CAFs (−CAF) or in the presence of Meso109F (+109F) or Meso125F (+125F) and treated with 3 or 10 μM cisplatin or vehicle. Micrographs were taken after 72 h and numbers of GFP^+^ tumor cells (TC) were determined by automated image analysis. GFP^+^ SPC212 (**E**) or GFP^+^ MSTO-211H (**F**) were cultured as monolayers in the absence of CAFs (−CAF) or in the presence of Meso109F (+109F) or Meso125F (+125F) and treated with 0.3, 1, 3 or 10 μM pemetrexed (Pem), the combination of 1 μM cisplatin (Cis) with pemetrexed (10 μM for SPC212, 1 μM for MSTO-211H) or vehicle (DMSO). Micrographs were taken after 72 h and numbers of GFP^+^ tumor cells (TC) were determined by automated image analysis. * *p* < 0.05, ** *p* < 0.01, *** *p* < 0.001 drug treated versus vehicle treated, ^#^*p* < 0.05, ^##^*p* < 0.01, ^###^*p* < 0.001 growth in presence of Meso-CAFs versus growth in absence of Meso-CAFs, ° *p* < 0.05, °° *p* < 0.01 percent inhibition in presence of Meso-CAFs versus percent inhibition in absence of Meso-CAFs, one-way ANOVA with Tukey’s multiple comparisons test

CAFs have been proposed to protect cancer cells from chemotherapy-induced effects [36, 37]. Therefore, we explored the impact of Meso-CAFs on the response of PM cells to cisplatin, a standard chemotherapeutic agent used in PM. However, co-cultivation of PM cells with Meso-CAFs did not show any protection of SPC212 or MSTO-211H cells against cisplatin by either Meso109F or Meso125F in either the 2D (Fig. 6A and B) or the 3D model (Fig. 6C and D). Pemetrexed is used in combination with cisplatin and was thus, also evaluated. SPC212 cells showed no significant inhibition by pemetrexed at all tested concentrations irrespective of Meso-CAF presence or absence (Fig. 6E). MSTO-211H cells in contrast, were significantly inhibited by pemetrexed compared to vehicle control at concentrations of 1 μM and above (Fig. 6F). The effect of pemetrexed on MSTO-211H was slightly reduced in co-culture with Meso-CAFs. Effects of the pemetrexed and cisplatin combination at the tested concentration were similar to those of cisplatin alone in the presence or absence of Meso-CAFs in both PM cell lines (Fig. 6E and F).

## Discussion

In several types of cancer including lung, breast, and colon cancer, CAFs have been extensively studied and shown to contribute to proliferation, migration, invasion, and drug resistance [38–40]. PM is characterized by rapid spreading along pleural surfaces, local invasion into surrounding tissues, poor response to therapies and a lack of oncogenic drivers, but few studies have investigated the role of CAFs in PM so far. Li et al. have shown that PM cell lines can stimulate MRC-5 lung fibroblasts to produce HGF, which in turn stimulated growth and migration of PM cells [41]. Ohara et al. reported that proliferation, migration, and invasion of PM cells was enhanced by the presence of normal human lung fibroblasts in a partly CTGF-dependent manner [42]. The same group also found in an immunohistochemistry study that CTGF expression in fibroblasts of PM specimens correlated with poor prognosis [43]. Recently, medium conditioned by cultures of the human lung fibroblasts MRC-5 and IMR-90 was shown to regulate the activity of multiple kinases in PM cells, especially those connected to the mitogen-activated protein kinase (MAPK) pathway [44]. To the best of our knowledge, this is the first study to characterize the transcript and protein expression patterns of patient-derived Meso-CAFs. Meso-CAFs express typical CAF markers such as α-SMA and PDGFR-α [45], while lacking expression of the PM marker MSLN as well as endothelial cell marker expression (not shown). Due to their mesenchymal origin, PM cells themselves show high expression of several markers previously used to identify CAFs such as FAP or VIM [2, 45], limiting the usefulness of these markers in PM. Our study, however, has identified a number of additional genes with strong expression differences between PM cells and Meso-CAFs. Several of these, like TPD52, TCF21, or ELN provide an excellent separation even between Meso-CAFs and cells from sarcomatoid PM. Although in our current study identified by bulk mRNA expression data from cultured cells, these genes represent promising candidates for further evaluation in immunohistochemistry studies of PM patients.

Interestingly, one of the Meso-CAFs (Meso109F) showed germline variants in FANCL and MSH3 and somatic mutations in EP300 and NTRK3. The FANCL variant has a relatively high incidence of up to 0.5% in the general population. Germline variants of DNA repair genes including in addition to BAP1 several members of the Fanconia anemia complementation group (FANC) family as well as MSH3 have been reported in PM [46, 47]. In the current case, it is tempting to speculate that combination of the two germline variants in DNA repair genes might have contributed to PM development and/or facilitated acquisition of the additional mutations in the Meso-CAFs. Nevertheless, the genetic alterations in Meso109F do not seem to have a major impact on their Meso-CAF phenotype, since their effect on PM cells was very similar to that of the other Meso-CAFs, which do not show any of the variants.

Our proteome analysis revealed that Meso-CAFs are not only clearly distinguishable from PM cells but also differ from normal lung fibroblasts. The strongest association of proteins upregulated in the secretome of Meso-CAFs was seen with ECM remodeling processes in agreement with the notion that production and remodeling of the ECM is a key feature of CAFs [48]. Moreover, multiple potent signaling molecules were identified in the secretomes of Meso-CAFs like the lymphangiogenesis inducer VEGFC [49] or the activin antagonist follistatin-like 3 (FSTL3) [50] that, in addition to directly affecting tumor cells, could have impacts on non-malignant cells in the TME and influence angiogenesis or immune responses. Dual immunotherapy with nivolumab and ipilimumab has recently become the first line therapy for non-epithelioid PM and is under discussion as first line therapy for epithelioid PM as well [8]. The novel data on Meso-CAF markers and the cell models presented in our study may prove to be useful for investigating the impact of Meso-CAFs on immune checkpoint expression in PM cells and/or immunotherapy response of PM patients.

It is generally acknowledged that CAFs are heterogeneous and multiple studies have identified different CAF subtypes within a given cancer type based on single cell transcriptomic data [9]. Future work will elucidate whether Meso-CAFs can also be divided into several subgroups that may differ with respect to expression profiles and functionalities. In the current study, we compared secreted proteins of cultured Meso-CAFs with secreted proteins of CAF cultures from other cancer types. On the one hand, in the Venn diagram analysis 207 of the 495 secreted proteins of Meso-CAFs were shared by all CAF types included (breast, lung, colon). On the other hand, more than 100 proteins secreted by Meso-CAFs had not been identified in the secretome of any of the other CAF types. It should be considered, that methodical differences between the studies from which the data were extracted could have contributed to this result. Nevertheless, these data might indicate either pre-existing differences in the cells from which the different CAFs originate or different activation profiles induced by different types of cancer cells. Single cell data have recently begun to unravel the normal and perturbed-state fibroblast lineages of mice and humans [51]. Interestingly, it was found that many lung fibroblast-specific genes were downregulated in lung CAFs [52], suggesting that CAFs from different tumors might be less variable than the respective unperturbed normal fibroblast populations in healthy organs.

With respect to growth and migration of PM cells, our data demonstrate a strong stimulating effect of Meso-CAFs, which is in agreement with previous reports, for instance in colon cancer [40] or lung cancer [39]. For the inhibitor treatment experiments, we decided to use co-cultures of Meso-CAFs and PM cells rather than Meso-CAF-CM, because co-culturing better reflects an in vivo treatment situation, in which Meso-CAFs provide a continuous supply of secreted factors and both Meso-CAFs and PM cells are exposed to the inhibitors. The model implies, however, that inhibitors may not only modulate the response of PM cells to signals from Meso-CAFs but could also modulate the secretion of signaling molecules by Meso-CAFs. Using this co-culture model and a panel of pathway inhibitors, we identified two signaling pathways, c-Met/PI3K and WNT, as critical for Meso-CAF-mediated growth stimulation of PM cells. C-Met is the tyrosine kinase receptor for the growth factor HGF, which is highly expressed in Meso-CAFs but not in PM cells. It can signal via the PI3K and the MAPK pathway [53], and blocking the PI3K pathway with LY294002 (but not inhibition of the MAPK pathway by U0126) had a similar effect as the c-Met inhibitor crizotinib in our models. This suggests that HGF (Meso-CAF) to c-Met (PM) signaling entertains the PI3K pathway in PM cells resulting in increased PM cell growth. Our data on crizotinib are in good agreement with the data from Li et al. using an HGF antibody or the HGF antagonist NK4 to inhibit the growth promoting effect of fibroblasts on PM cells [41]. C-Met and PI3K have been suggested as targets for PM therapy [54, 55]. According to our data, both would be suitable targets to block growth-stimulating effects of Meso-CAFs.

Aberrant WNT signaling is a key oncogenic driver in colon cancer, mostly as a consequence of mutations in Adenomatous Polyposis Coli (APC), β-catenin or axin [56]. In PM, similar mutations have not been identified but several WNT pathway related molecules were found overexpressed [57]. Our data demonstrate the importance of WNT signaling for growth stimulation of PM cells by Meso-CAFs. WNT signaling can activate canonical and non-canonical signaling pathways [58]. The WNT inhibitor (WNT-C59) used in our study targets porcupine (PORCN), which is required for secretion and activation of WNTs [59]. Hence, WNT-C59 affects both, canonical and non-canonical pathways. WNT5A and WNT5B, the two WNTs identified in Meso-CAF secretomes have been primarily linked to non-canonical signaling [60]. WNT5A was recently detected in colon CAFs and linked to colon cancer progression [61]. In another study using colon CAFs, it was shown that canonical signaling via WNT2 enhances tumor progression by autocrine stimulation of fibroblasts and by inducing angiogenesis [27, 40]. In our study, however, Meso-CAFs expressed much lower levels of WNT2 mRNA compared to the colon CAFs used as controls (CAF-3), and WNT2 protein was not detected in the secretome of Meso-CAFs. With respect to therapeutic approaches, several studies have reported anti-tumor and chemosensitizing effects of WNT inhibition in PM cells [62, 63] and our data clearly support further evaluation of WNT inhibition as part of PM therapy.

Although CAFs have been frequently shown to promote chemoresistance of tumor cells [36, 37] the presence of Meso-CAFs did not provide any protection of PM cells during cisplatin treatment in our study. Since cisplatin mainly targets proliferating cells, the Meso-CAF-mediated induction of PM cell proliferation may have antagonized potentially protective signals secreted by the fibroblasts. These data suggest that Meso-CAFs may not be responsible for cisplatin resistance in PM patients and might even contribute to the effectiveness of cisplatin-based regimens via stimulating PM cell proliferation. Only one of the two PM cell lines tested was sensitive to pemetrexed and hence, the moderate protective effect of Meso-CAFs against pemetrexed treatment seen in this cell line has to be interpreted with caution. The results indicate, however, that the impact of Meso-CAFs on drug response are highly drug-dependent. This is further exemplified by the finding that Meso-CAFs showed a significant protective effect when co-cultures were treated with the EGFR inhibitor erlotinib. Recently, it was shown that different subtypes of lung CAFs influence the efficacy of the EGFR inhibitor osimertinib [64]. Robust protection was found for lung CAFs expressing high levels of HGF and FGF7, both also highly expressed by Meso-CAFs. This could, at least in part, explain the poor response to EGFR-targeting therapies in PM [65]. The data on cisplatin and erlotinib show that Meso-CAFs may differentially influence treatment outcomes depending on the specific mechanisms of action of the therapeutic agent.

## Conclusions

Taken together, our study strongly suggests that Meso-CAFs have a significant impact on PM progression and sheds light on the signaling pathways responsible for their growth promoting function. The demonstrated data may contribute to a better understanding of the aggressiveness and therapy response of PM and could help to develop novel therapeutic strategies.

## Supplementary Information

**Additional file 1: Supplementary Methods.** Extended methodologic description of the proteomic analysis.

**Additional file 2: Supplementary Table S1.** Overview of cell types used in the study. **Supplementary Table S2.** List of primers used for qRT-PCR and evaluation of germline DNA mutations. **Supplementary Table S3.** Genes investigated by next generation sequencing in the TruSight Oncology 500 panel. **Supplementary Table S4.** Differentially expressed genes of Meso-CAFs compared to PM cells. **Supplementary Table S5.** Genes with high fold change in Meso-CAFs compared to CAF-3. **Supplementary Table S6.** Numbers of identified proteins in the nuclear, cytoplasmic and supernatant fractions of Meso-CAFs and NLFs. **Supplementary Table S7.** Identified proteins of Meso-CAFs in the nuclear, cytoplasmic and supernatant fraction. **Supplementary Table S8.** Identified proteins of NLFs in the nuclear, cytoplasmic and supernatant fraction. **Supplementary Table S9.** Actively secreted proteins exclusively identified in Meso-CAFs or significantly upregulated compared to NLFs.

**Additional file 3: Supplementary Fig. S1.** Cell morphology of Meso-CAFs and PM cells. **Supplementary Fig. S2.** Genomic profiles of CAFs and PM cells. **Supplementary Fig. S3**. Immunohistochemistry of Meso-CAFs and PM cells. **Supplementary Fig. S4**. Sanger sequencing of Meso109F and corresponding whole blood. **Supplementary Fig. S5.** Unsupervised clustering of CAFs and PM cells. **Supplementary Fig. S6.** Differentially expressed genes in Meso-CAFs versus sarcomatoid PM. **Supplementary Fig. S7.** Volcano plots of differentially expressed proteins in Meso-CAFs versus NLFs. **Supplementary Fig. S8.** Biological processes associated with proteins differentially expressed between Meso-CAFs and NLFs. **Supplementary Fig. S9.** Biological processes associated with secreted proteins found in CAFs from multiple cancers versus only in Meso-CAFs. **Supplementary Fig. S10.** Presence or absence of selected proteins from the secretome of Meso-CAFs in the secretomes of CAFs from other cancers. **Supplementary Fig. S11.** Migration parameters of PM cells in the presence or absence of Meso-CAFs. **Supplementary Fig. S12.** Stimulation of MSTO-211H growth by VMC59F. **Supplementary Fig. S13.** Growth- and migration promoting effects of CM from Meso-CAFs versus NLFs. **Supplementary Fig. S14.** Absence of inhibitor-induced cytotoxicity in Meso-CAFs. **Supplementary Fig. S15.** Response of PM cells to signaling pathway inhibitors in the presence or absence of Meso-CAFs. **Supplementary Fig. S16.** Time course of SPC212 cell response to inhibitors in the presence or absence of Meso-CAFs. **Supplementary Fig. S17.** Time course of MSTO-211H cell response to inhibitors in the presence or absence of Meso-CAFs.

## Data Availability

All datasets generated and analyzed during the current study are available in the following repositories under the respective identifiers. Proteomics Identifications Database (PRIDE) (https://www.ebi.ac.uk/pride): PXD035987, PXD036017, PXD036127; ArrayExpress (https://www.ebi.ac.uk/biostudies/arrayexpress): E-MTAB-12177, E-MTAB-8986 (Expression Microarray); E-MTAB-12179, E-MTAB-8987 (Array CGH).
