# Supplementary material for: Mesothelioma-associated fibroblasts enhance proliferation and migration of pleural mesothelioma cells via c-Met/PI3K and WNT signaling but do not protect against cisplatin
Source: J Exp Clin Cancer Res. 2023 Jan 23;42:27. doi: 10.1186/s13046-022-02582-0 (PMC9869633; doi:10.1186/s13046-022-02582-0)
Supplement: Supplementary file 1 — Additional file 1: Supplementary Methods. Extended methodologic description of the proteomic analysis. [file 13046_2022_2582_MOESM1_ESM.pdf]

Additional file 1: Supplementary Methods. Extended methodologic description of the proteomic analysis of Meso-CAFs and normal lung fibroblasts (NLFs)

Cellular fractionation

The nuclear, cytoplasmic and supernatant protein fractions for analysis were obtained as briefly indicated in the manuscript and described previously by Slany et al. [1]. Prior to protein extraction, cells were washed twice with sterile filtered 1 x PBS at 37 °C and cultivated for 6 h in 3 ml [for T25] or 6 ml [for T75] of the respective FCS-free medium to avoid protein contamination from serum. The secreted proteins were obtained by centrifugation (587 × g, 4°C, 5 min) of cell supernatants to remove cellular debris, followed by the precipitation of proteins with ice-cold ethanol overnight at -20°C. To isolate cytoplasmic proteins, cells were suspended in ice-cold lysis buffer (10 mM HEPES/NaOH (pH 7.4), 0.25 M sucrose, 10 mM NaCl, 3.5 mM $MgCl_2$, 0.5% Triton X-100, 1 mM EGTA) including a protease/phosphatase inhibitor cocktail (Sigma-Aldrich, St. Louis, MO, USA), harvested using a cell scraper and pressed through a 23 g syringe to induce full cell lysis. Cytoplasmic proteins were separated from nuclei by centrifugation (2300 × g, 4°C, 5 min) and precipitated in ice-cold ethanol at-20°C overnight. To obtain nuclear extracts, the remaining pellets were lysed on ice with a hypertonic solution (10 mM Tris/HCl (pH 7.4), 1 mM EDTA (pH 7.5), 0.5 M NaCl) and subsequently treated with NP-40 buffer (10 mM Tris/HCl (pH 7.4), 1 mM EDTA (pH 7.5), 0.5% NP-40) containing the protease/phosphatase inhibitor cocktail. Centrifugation (2300 × g, 4°C, 5 min) separated the nuclear fraction from the remaining cellular components, and the nuclear proteins were precipitated with ethanol at -20°C overnight. The precipitated proteins of all fractions were pelleted, dried under vacuum and dissolved in sample buffer (7.5 M urea, 1.5 M thiourea, 4% CHAPS, 0.05% sodium dodecyl sulphate (SDS), 100 mM dithiothreitol (DDT)), and the protein concentrations were assessed using a Bradford assay (Bio-Rad Laboratories, Hercules, CA, USA).

Digestion of proteins (MS sample preparation)

Enzymatic digestion of proteins into peptides was performed based on the in-solution digestion protocol from Humphrey *et al*. 2018 [2] with minor changes. Protein (25 µg per sample) was precipitated overnight with ice-cold ethanol to remove SDS, centrifuged and washed with 80% ethanol. After further centrifugation, the samples were dried, dissolved in 90 µl of 4% sodium deoxycholate buffer containing 100 mM Tris-HCl (pH 8.0) and heat-treated for 5 min (95°C). The proteins were reduced with 100 mM Tris(2-carboxyethyl)phosphine (pH 8.0), alkylated with 400 mM 2-chloroacetamide (pH 8.0) and enzymatically digested twice at 37°C using a trypsin/lys-c mixture (Promega Corporation, Fitchburg, WI, USA) with a total incubation time of 20 h. Samples were cleaned-up on SDB-RPS StageTips according to Humphrey et al. 2018 [2], and peptides were eluted with a 60% acetonitrile solution containing 0.5% of a 28% ammonium hydroxide solution (Sigma-Aldrich, St. Louis, MO, USA). The elutes were dried by vacuum centrifugation and stored at -20°C until MS analysis.

TimsTOF data acquisition

The dried peptides were dissolved in 5 µl of 30% formic acid (FA) containing four synthetic standard peptides (10 fmol each) and diluted with 40 µl of loading solvent (97.9% H_2O, 2% acetonitrile (ACN), 0.05% trifluoroacetic acid). The peptide samples were subjected to LC-MS/MS analyses on a Dionex Ultimate 3000 nano LC-system (Thermo Scientific, Thermo Fisher Scientific, Carlsbad, CA, USA) coupled to a timsTOF pro mass spectrometer (Bruker Daltonics, Bruker Corporation, Billerica, MA, USA). Each biological sample was recorded in technical duplicates and 5 µL per sample were injected for analysis. The samples were first loaded on a 2 cm x 100 µm C18 Pepmap100 pre-column (Thermo Scientific, Thermo Fisher Scientific, Carlsbad, CA, USA) at a flow rate of 10 µl/min using mobile phase A (99.9% H_2O, 0.1% FA) and then eluted from the pre-column to a 25 cm x 75 µm Aurora Series emitter column (IonOpticks, Fitzroy, VIC, Australia) at a flow rate of 300 nl/min. Separation was achieved using a gradient of 8% to 40% mobile phase B (79.9% ACN, 20% H_2O, 0.1% FA) over 55 min for supernatants and over 90 min for cytoplasmic and nuclear fractions, resulting

in total LC run times of 85 and 135 min, respectively, including washing and equilibration steps. MS analyses were accomplished using a timsTOF Pro mass spectrometer equipped with a captive spray ion source run at 1650 V. The timsTOF Pro was operated in Parallel Accumulation-Serial Fragmentation (PASEF) mode. Trapped ion mobility separation was achieved by applying a 1/k0 scan range from 0.60 to 1.60 V.s/cm2.

Data analysis

Protein identification and label-free quantification was performed using MaxQuant 1.6.17.0 [3] and employing the Andromeda software searching against the UniProt Database for human proteins (version 12/2019 with 20,380 entries). Parameters were set in a way to allow a mass tolerance of 20 ppm for MS1 spectra and 40 ppm for MS/MS spectra, and to restrict the FDR to ≤ 0.01 and the number of missed cleavages to a maximum of two. Search criteria also included carbamidomethylation of cysteines as fixed modification and methionine oxidation as well as N-terminal protein acetylation as variable modifications. For a positive identification, at least two peptides in case of cytoplasmic and nuclear proteins, and one peptide for secreted proteins, together with at least one unique peptide in all cases, had to be detected. "Match between runs" was enabled, setting the match ion mobility window to 0.1, the match time window to 0.7 min, and the alignment time window to 20 min.

Statistical evaluation was mainly performed using Perseus software (version 1.6.14.0) [4, 5]. We initially removed reverse sequences, potential contaminants as well as proteins identified only by site, followed by a log_2-transformation of the label-free quantification (LFQ) values. The technical replicates were averaged and the protein groups were filtered for valid values, keeping only those identified in at least 2 biological replicates of one cell type. Missing values were replaced from a normal distribution in order to enable t-testing and determine proteins significantly regulated between different cell types. In addition, data of Meso-CAFs and NLFs was also processed separately as described above and resulting protein lists of both groups were compared using Venny 2.1 [6] to clearly identify exclusively expressed proteins by one group and rule out a loss of information during imputation. The data analysis was also

conducted in R [7] using the package “DEP” [8] following the same processing steps as described above, and a heatmap over the LFQ values of all proteins in the supernatant was generated without averaging the technical replicates.

References

1. Slany A, Paulitschke V, Haudek-Prinz V, Meshcheryakova A, Gerner C. Determination of cell type-specific proteome signatures of primary human leukocytes, endothelial cells, keratinocytes, hepatocytes, fibroblasts and melanocytes by comparative proteome profiling. Electrophoresis. 2014;35:1428-38.
2. Humphrey SJ, Karayel O, James DE, Mann M. High-throughput and high-sensitivity phosphoproteomics with the EasyPhos platform. Nat Protoc. 2018;13:1897-916.
3. Tyanova S, Temu T, Cox J. The MaxQuant computational platform for mass spectrometry-based shotgun proteomics. Nat Protoc. 2016;11:2301-19.
4. Cox J, Mann M. MaxQuant enables high peptide identification rates, individualized p.p.b.-range mass accuracies and proteome-wide protein quantification. Nat Biotechnol. 2008;26:1367-72.
5. Cox J, Mann M. 1D and 2D annotation enrichment: a statistical method integrating quantitative proteomics with complementary high-throughput data. BMC Bioinformatics. 2012;13 Suppl 16:S12.
6. Oliveros JC. Venny. An interactive tool for comparing lists with Venn's diagrams. 2007-2015.
7. R Core Team. R: A language and environment for statistical computing. R Foundation for Statistical Computing, Vienna, Austria. 2018.
8. Zhang X, Smits AH, van Tilburg GB, Ovaa H, Huber W, Vermeulen M. Proteome-wide identification of ubiquitin interactions using UbIA-MS. Nat Protoc. 2018;13:530-50.
