# Supplementary material for: Mesothelioma-associated fibroblasts enhance proliferation and migration of pleural mesothelioma cells via c-Met/PI3K and WNT signaling but do not protect against cisplatin
Source: J Exp Clin Cancer Res. 2023 Jan 23;42:27. doi: 10.1186/s13046-022-02582-0 (PMC9869633; doi:10.1186/s13046-022-02582-0)
Supplement: Supplementary file 3 — Additional file 3: Supplementary Fig. S1. Cell morphology of Meso-CAFs and PM cells. Supplementary Fig. S2. Genomic profiles of CAFs and PM cells. Supplementary Fig. S3. Immunohistochemistry of Meso-CAFs and PM cells. Supplementary Fig. S4. Sanger sequencing of Meso109F and corresponding whole blood. Supplementary Fig. S5. Unsupervised clustering of CAFs and PM cells. Supplementary Fig. S6. Differentially expressed genes in Meso-CAFs versus sarcomatoid PM. Supplementary Fig. S7. Volcano plots of differentially expressed proteins in Meso-CAFs versus NLFs. Supplementary Fig. S8. Biological processes associated with proteins differentially expressed between Meso-CAFs and NLFs. Supplementary Fig. S9. Biological processes associated with secreted proteins found in CAFs from multiple cancers versus only in Meso-CAFs. Supplementary Fig. S10. Presence or absence of selected proteins from the secretome of Meso-CAFs in the secretomes of CAFs from other cancers. Supplementary Fig. S11. Migration parameters of PM cells in the presence or absence of Meso-CAFs. Supplementary Fig. S12. Stimulation of MSTO-211H growth by VMC59F. Supplementary Fig. S13. Growth- and migration promoting effects of CM from Meso-CAFs versus NLFs. Supplementary Fig. S14. Absence of inhibitor-induced cytotoxicity in Meso-CAFs. Supplementary Fig. S15. Response of PM cells to signaling pathway inhibitors in the presence or absence of Meso-CAFs. Supplementary Fig. S16. Time course of SPC212 cell response to inhibitors in the presence or absence of Meso-CAFs. Supplementary Fig. S17. Time course of MSTO-211H cell response to inhibitors in the presence or absence of Meso-CAFs. [file 13046_2022_2582_MOESM3_ESM.pdf]

Supplementary Figures

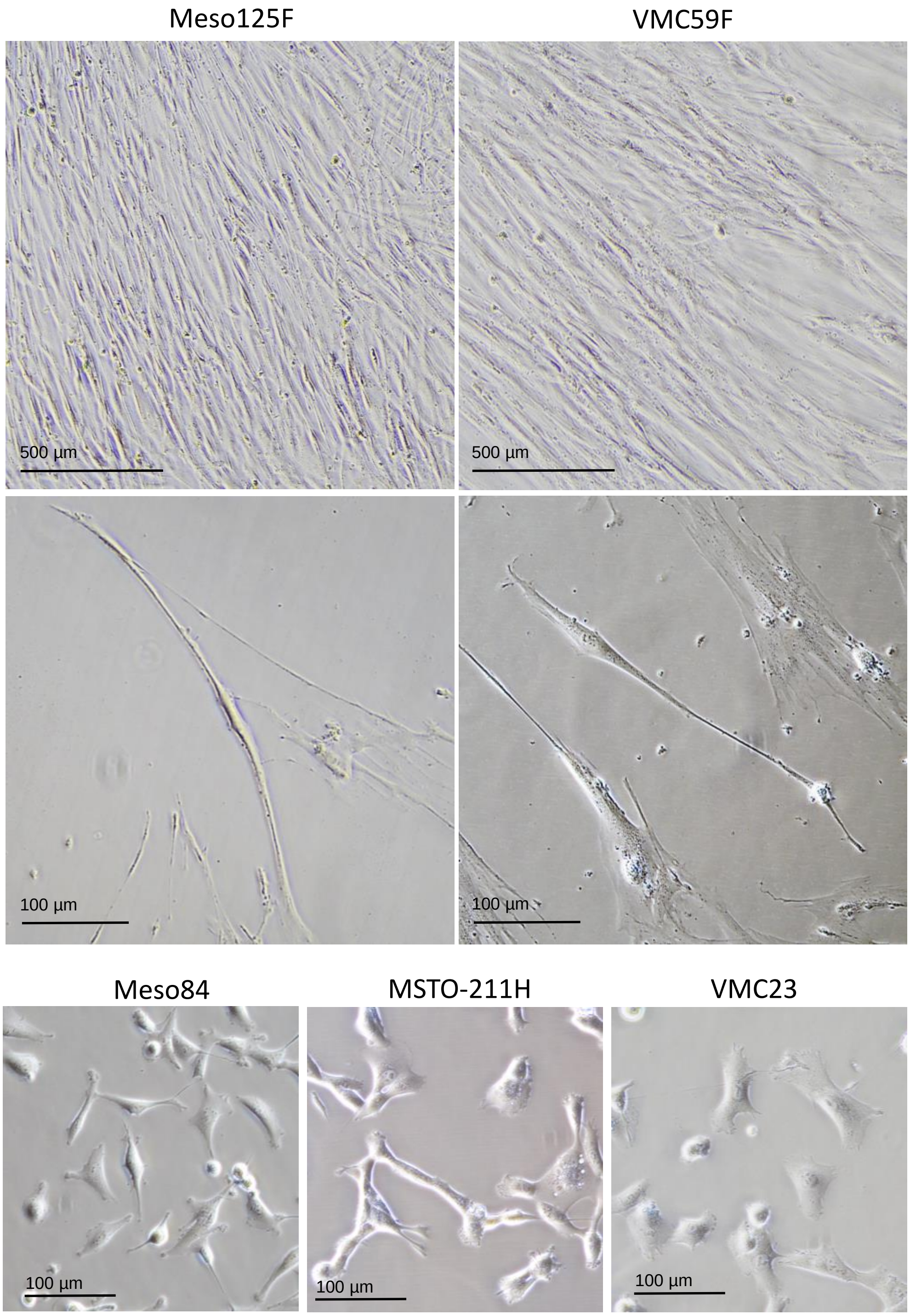

Supplementary Figure S1. Cell morphology of Meso-CAFs and PM cells. Micrographs of cultured Meso125F and VMC59F were taken at high (upper panels) and low (lower panels) cell density. Micrographs of Meso84 derived from sarcomatoid PM, MSTO-211H derived from biphasic PM and VMC23 derived from epithelioid PM were taken at low density.

Meso125F

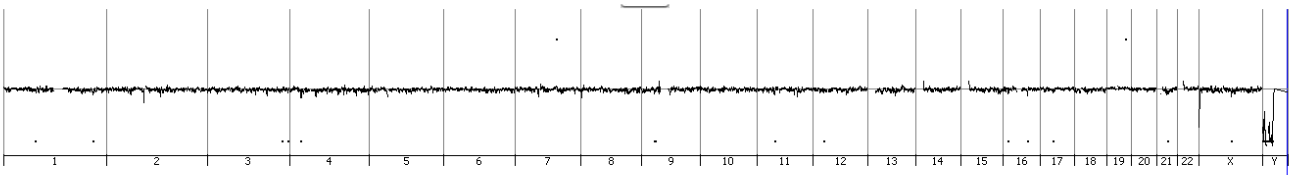

VMC59F

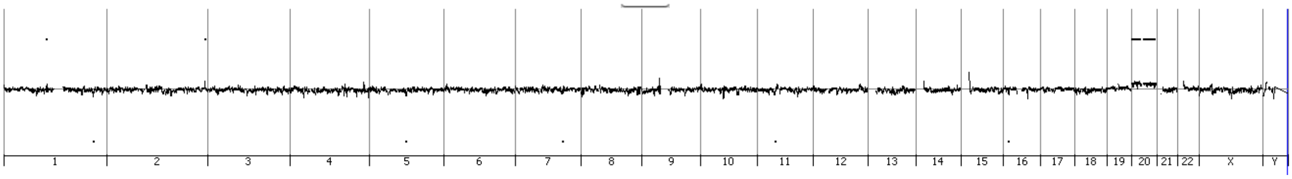

CAF-3

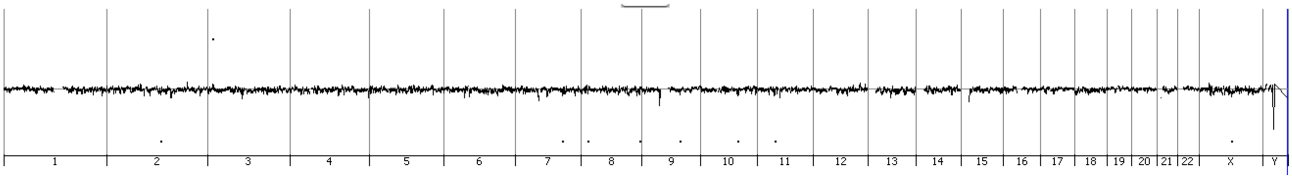

Meso84

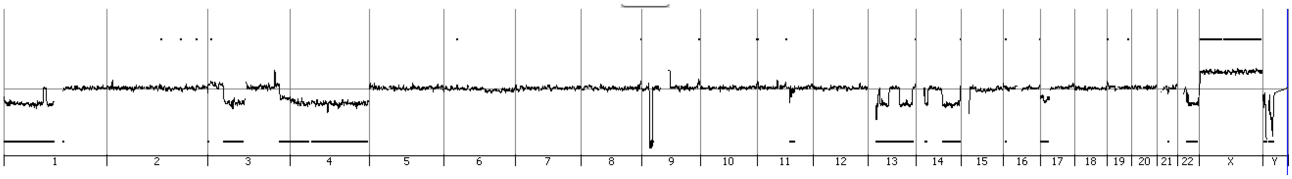

VMC23

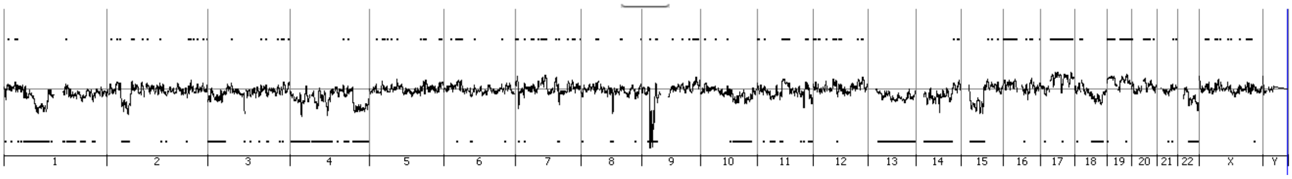

Supplementary Figure S2. Genomic profiles of CAFs and PM cells. Comparative genomic hybridization array (array CGH) profiles were established from Meso-CAFs (Meso125F, VMC59F), colon CAFs (CAF-3) and PM cell lines (Meso84, VMC23).

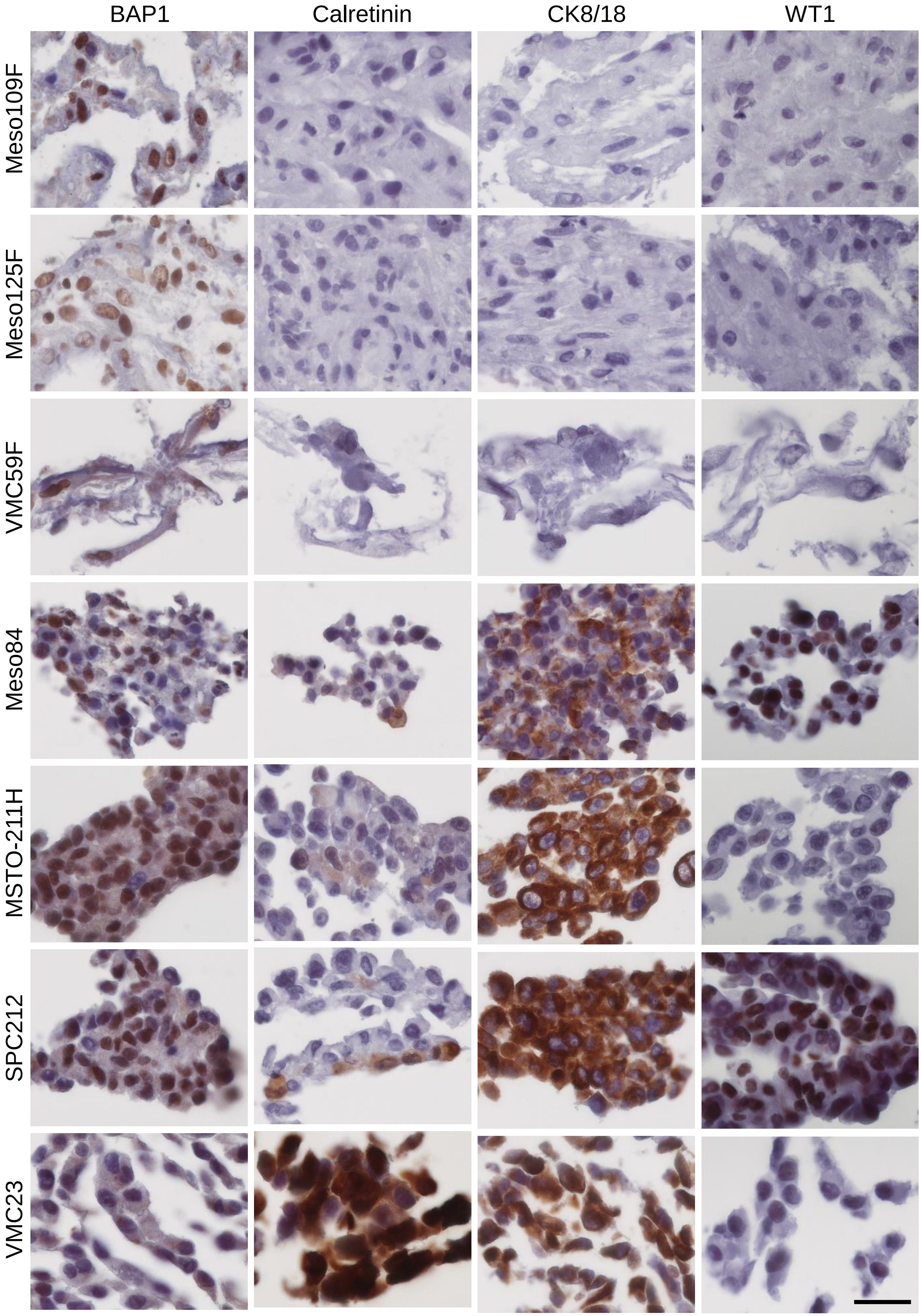
BAP1
Calretinin
CK8/18
WT1
Meso109F
Meso125F
VMC59F
Meso84
MSTO-211H
SPC212
VMC23

Supplementary Figure S3. Immunohistochemistry of Meso-CAFs and PM cells. Cells were formalin-fixed and paraffin-embedded, and sections were stained for the indicated antigens. Representative micrographs are shown. Scale bar = 25 µm.

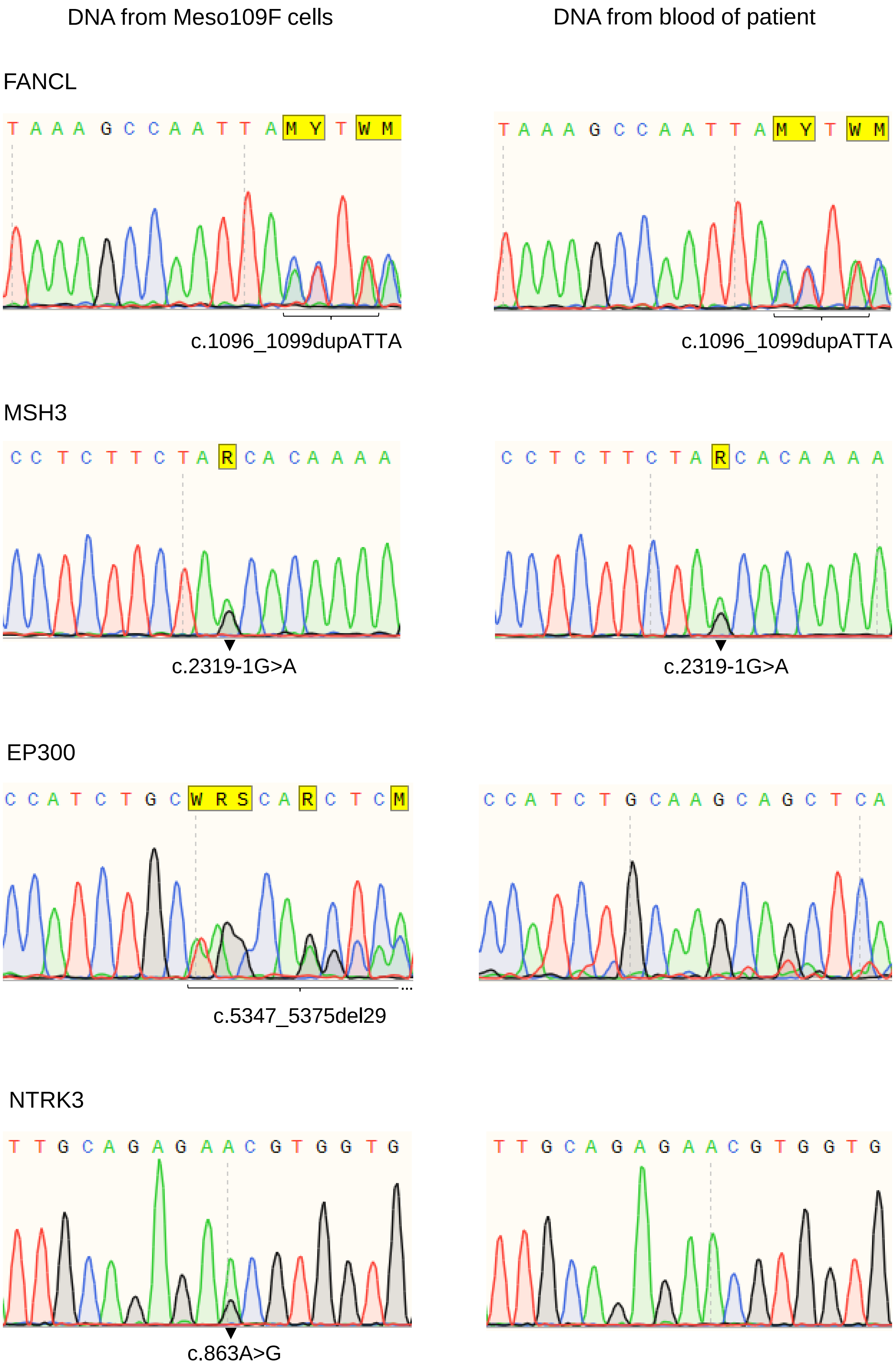
DNA from Meso109F cells
DNA from blood of patient
FANCL
T A A A G C C A A T T A M Y T W M
c.1096_1099dupATTA
T A A A G C C A A T T A M Y T W M
c.1096_1099dupATTA
MSH3
C C T C T T C T A R C A C A A A A
c.2319-1G>A
C C T C T T C T A R C A C A A A A
c.2319-1G>A
EP300
C C A T C T G C W R S C A R C T C M
c.5347_5375del29
C C A T C T G C A A G C A G C T C A
NTRK3
T T G C A G A G A A C G T G G T G
c.863A>G
T T G C A G A G A A C G T G G T G

Supplementary Figure S4. Sanger sequencing of Meso109F cells and corresponding whole blood. Loci in the indicated genes were amplified by PCR and Sanger sequenced. The corresponding genomic regions are shown for DNA isolated from Meso109F (left panels) and DNA isolated from whole blood of the patient from whom Meso109F was derived (right panels). Variants are indicated and can be recognized by overlapping peaks in the chromatograms.

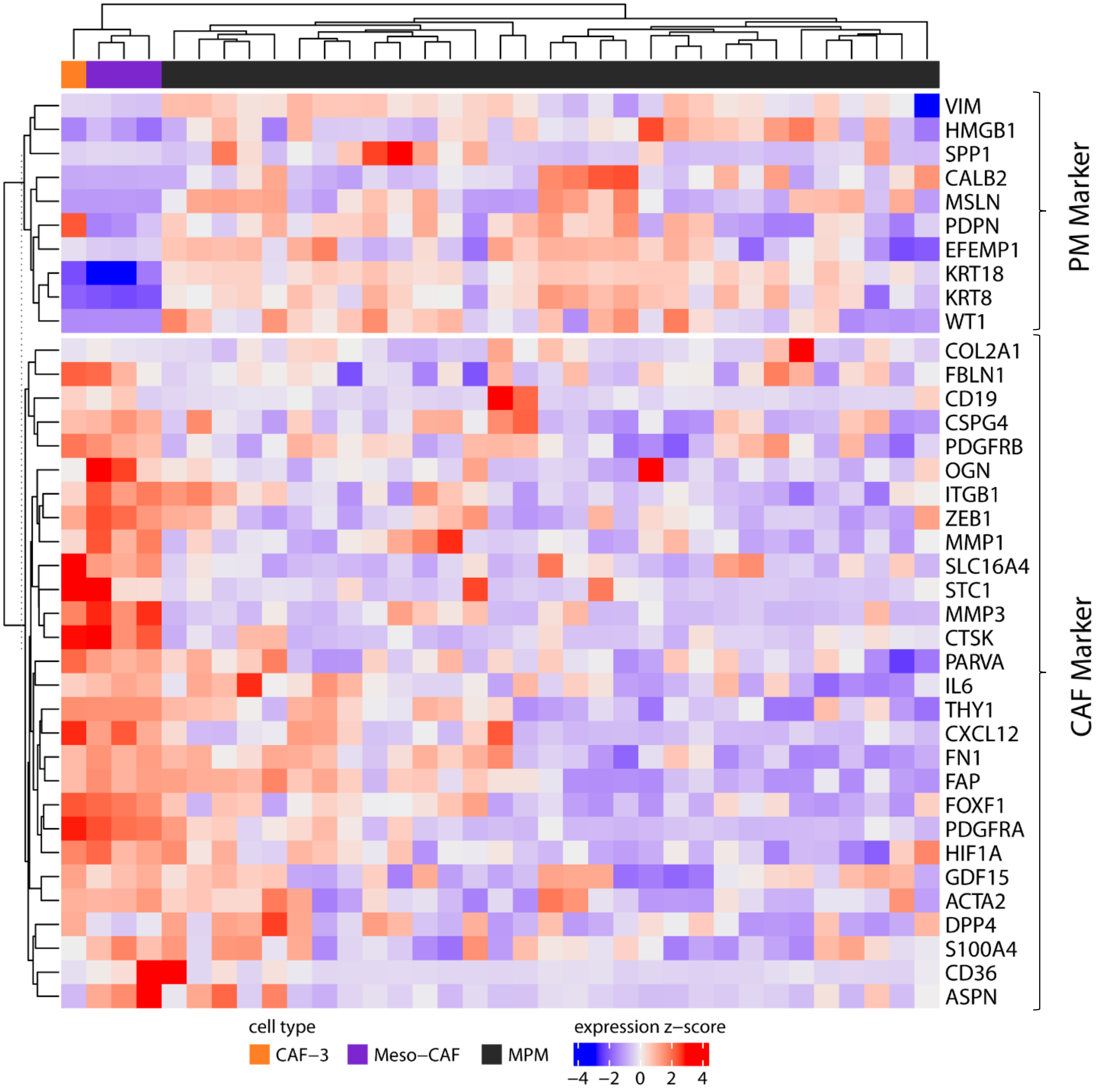

Supplementary Figure S5. Unsupervised clustering of CAFs and PM cells. Transcriptomic data of PM cell lines (n=31), Meso-CAFs (Meso109F, Meso125F, VMC59F) and colon CAFs (CAF-3) were used for unsupervised clustering using a list of PM and CAF marker genes.

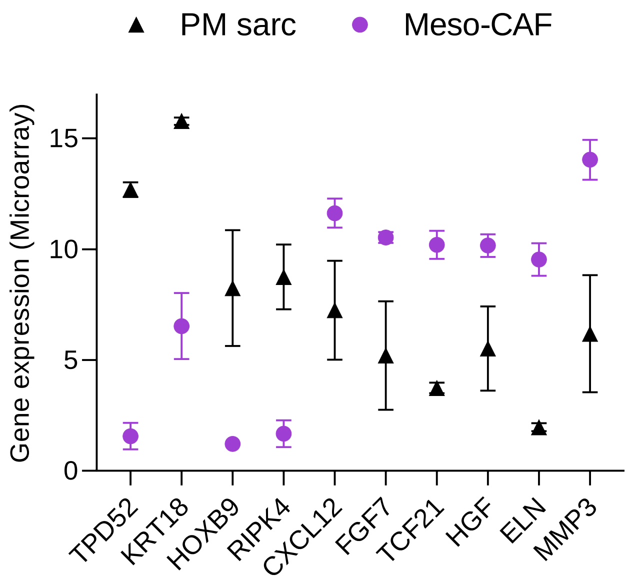

Supplementary Figure S6. Differentially expressed genes in Meso-CAFs versus sarcomatoid PM. Expression levels (log_2 transformed hybridization signals) of genes selected from whole genome gene expression microarrays are shown as means and SEM of 3 Meso-CAFs and 3 PM cell lines from sarcomatoid PM.

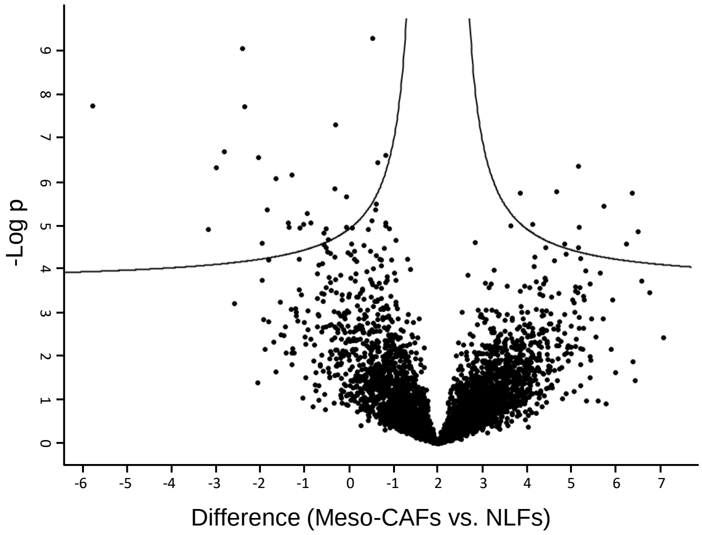

Nuclear fraction

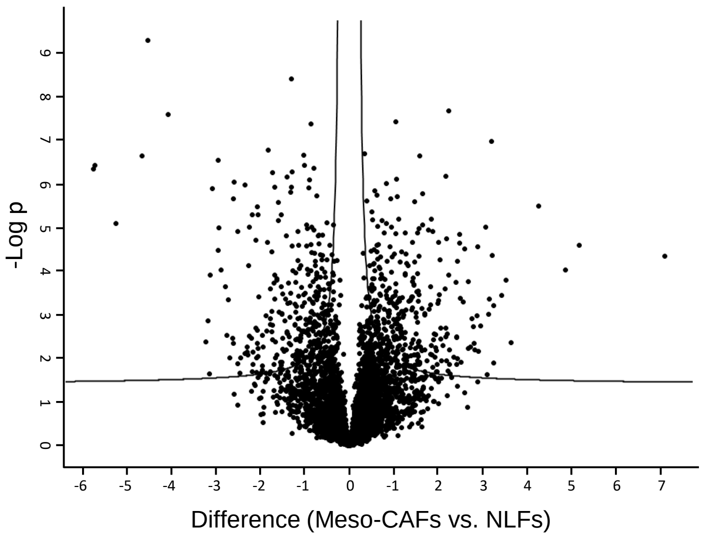

Cytoplasmic fraction

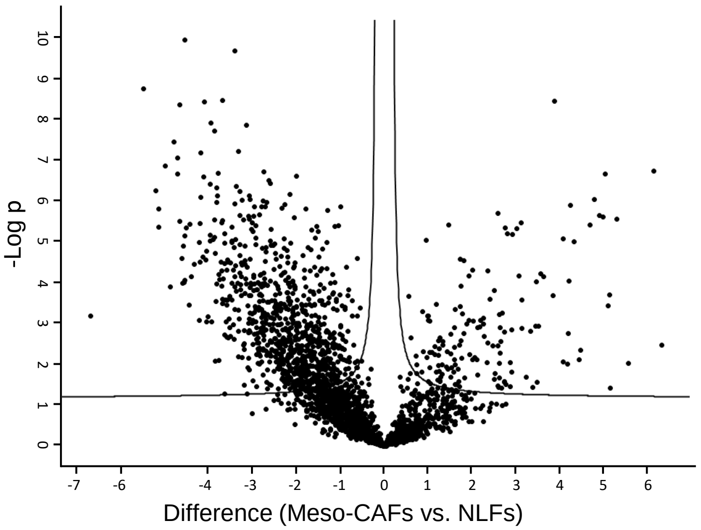

Supernatant fraction

Supplementary Figure S7. Volcano plots of differentially expressed proteins in Meso-CAFs versus normal lung fibroblasts (NLFs).

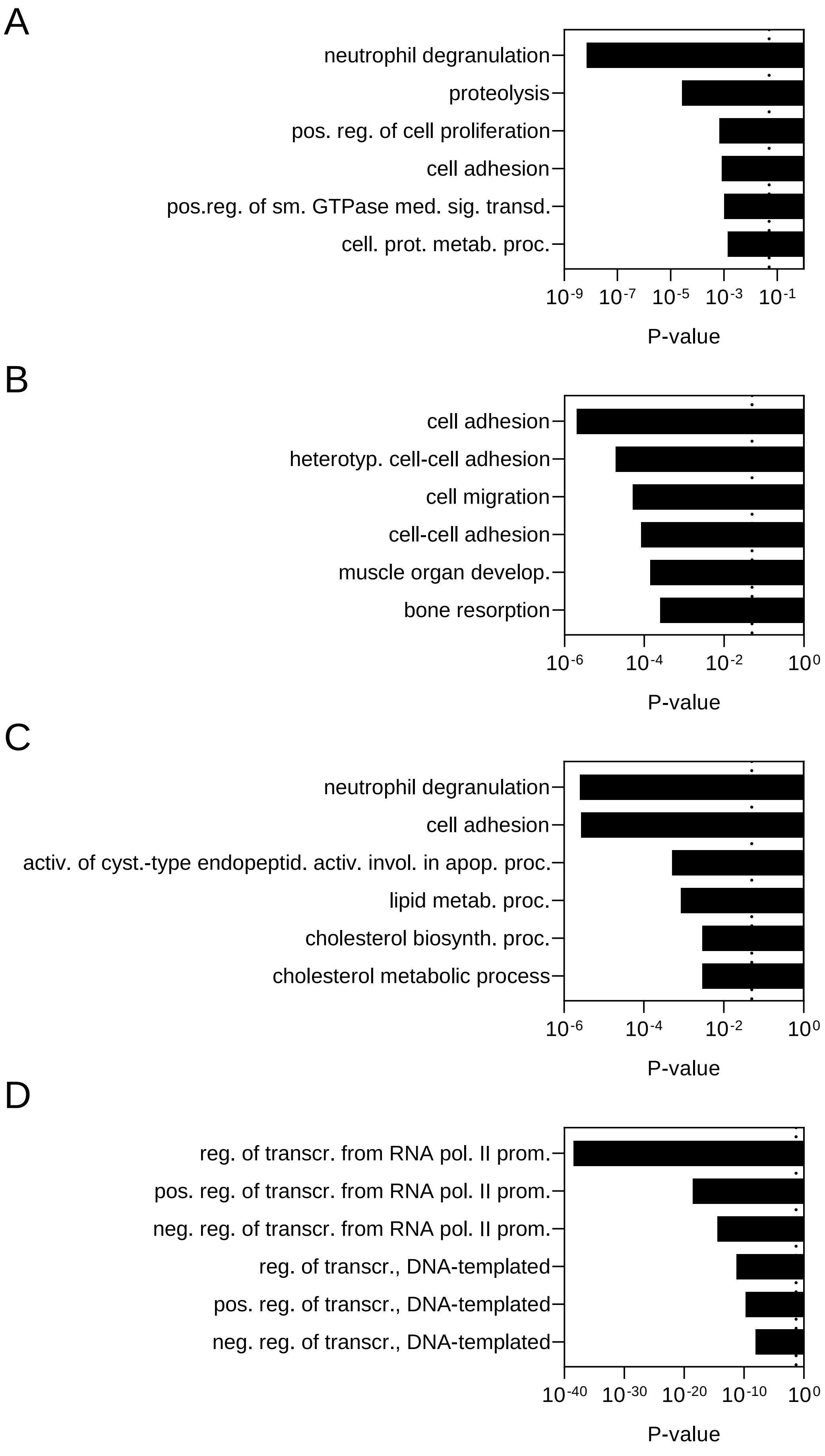

A
neutrophil degranulation
proteolysis
pos. reg. of cell proliferation
cell adhesion
pos.reg. of sm. GTPase med. sig. transd.
cell. prot. metab. proc.
10-9 10-7 10-5 10-3 10-1
P-value
B
cell adhesion
heterotyp. cell-cell adhesion
cell migration
cell-cell adhesion
muscle organ develop.
bone resorption
10-6 10-4 10-2 100
P-value
C
neutrophil degranulation
cell adhesion
activ. of cyst.-type endopeptid. activ. invol. in apop. proc.
lipid metab. proc.
cholesterol biosynth. proc.
cholesterol metabolic process
10-6 10-4 10-2 100
P-value
D
reg. of transcr. from RNA pol. II prom.
pos. reg. of transcr. from RNA pol. II prom.
neg. reg. of transcr. from RNA pol. II prom.
reg. of transcr., DNA-templated
pos. reg. of transcr., DNA-templated
neg. reg. of transcr., DNA-templated
10-40 10-30 10-20 10-10 100
P-value

Supplementary Figure S8. Biological processes associated with proteins differentially expressed between Meso-CAFs and normal lung fibroblasts (NLFs). (A) Biological processes associated with secreted proteins identified only in NLFs or significantly upregulated in NLFs compared to Meso-CAFs. (B) Biological processes associated with membrane proteins identified only in Meso-CAFs or significantly upregulated in Meso-CAFs compared to NLFs. (C) Biological processes associated with membrane proteins identified only in NLFs or significantly upregulated in NLFs compared to Meso-CAFs. (D) Biological processes associated with DNA-binding proteins identified only in Meso-CAFs or significantly upregulated in Meso-CAFs compared to NLFs. Due to the small number of DNA-binding proteins identified only in NLFs or significantly downregulated in NLFs compared to Meso-CAFs, no analysis was performed for this category.

A

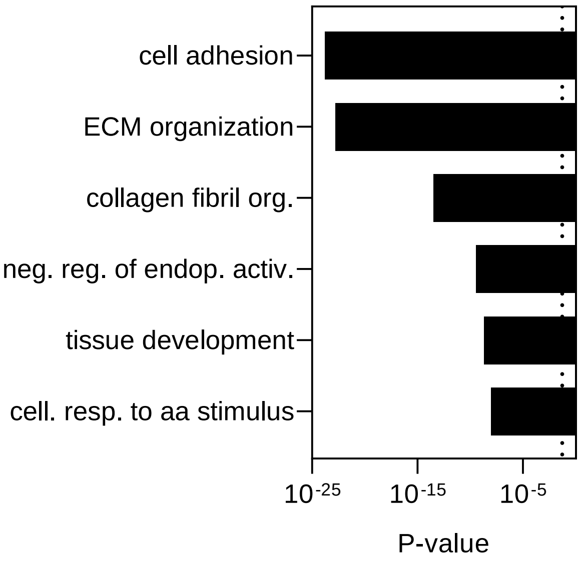

B

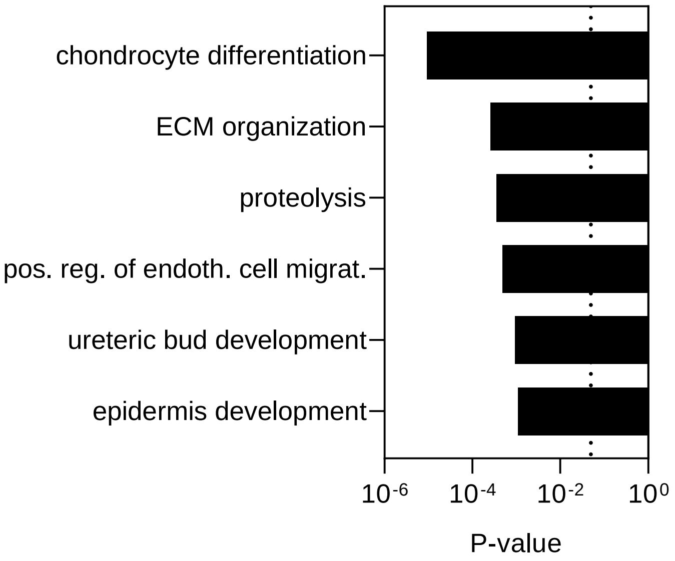

Supplementary Figure S9. Biological processes associated with secreted proteins found in CAFs from multiple cancers versus only in Meso-CAFs. (A) Biological processes associated with secreted proteins common to CAFs from PM, lung cancer, colon cancer and breast cancer. (B) Biological processes associated with secreted proteins found only in Meso-CAFs.

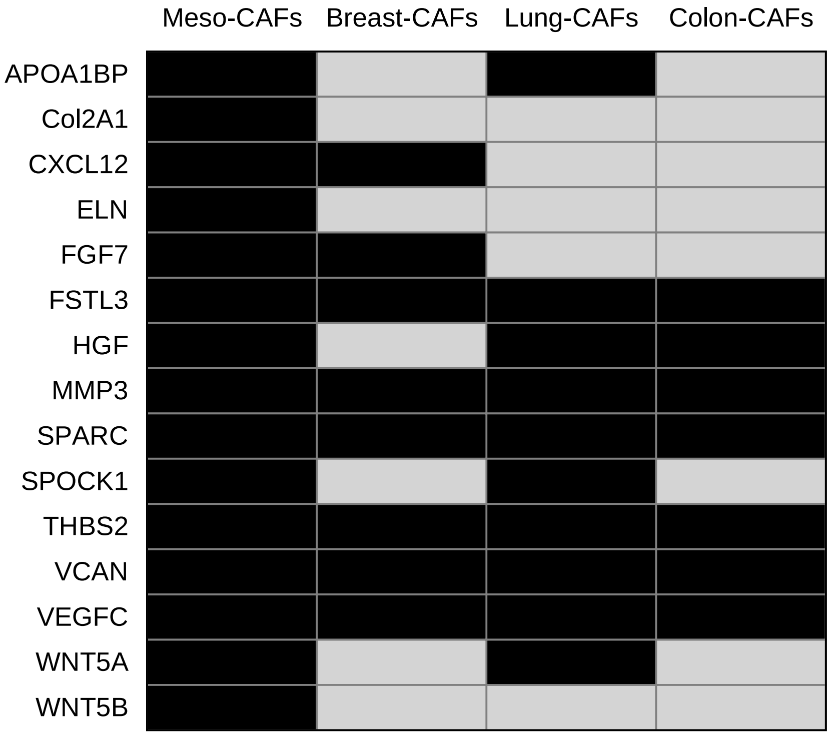

Supplementary Figure S10. Presence (black) or absence (gray) of selected proteins from the secretome of Meso-CAFs in the secretomes of CAFs from other cancers.

SPC212

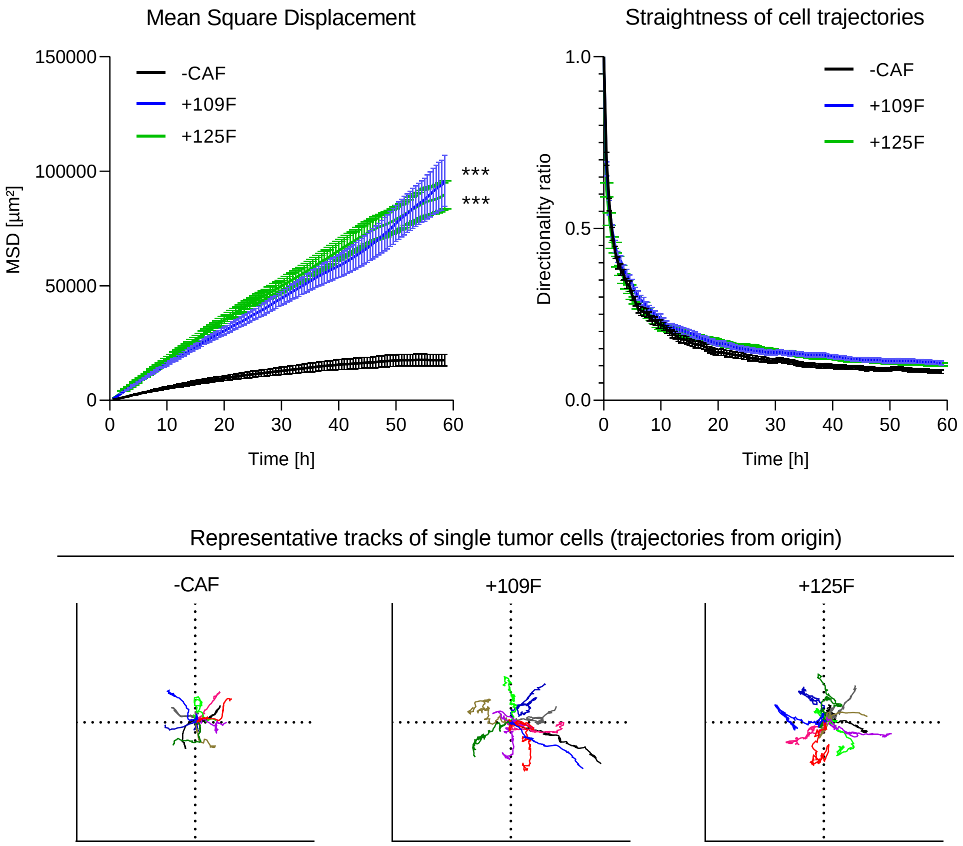

MSTO-211H

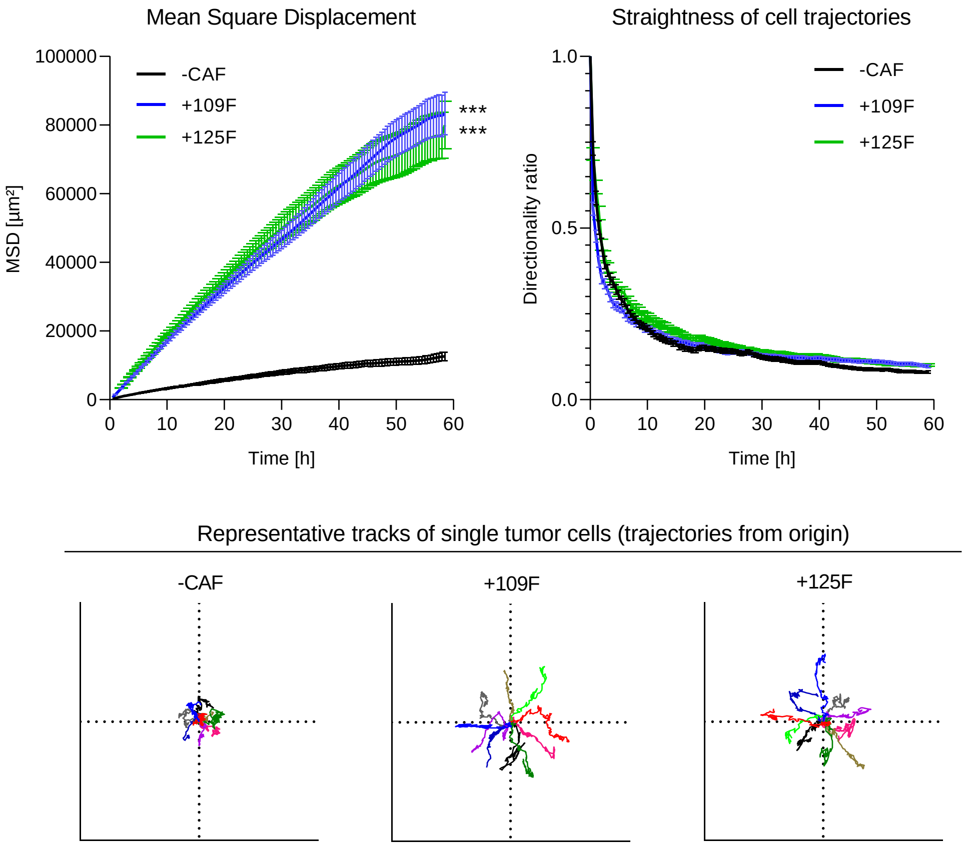

Supplementary Figure S11. Migration parameters of PM cells in the presence or absence of Meso-CAFs. GFP^+ SPC212 and GFP^+ MSTO-211H were cultured in the absence of CAFs (-CAF) or in the presence of Meso109F (+109F) or Meso125F (+125F). Individual tumor cells (n > 190 per condition) from three biological replicates were manually tracked for 60 h. Data for mean square displacement (MSD), straightness of cell trajectories and origin plots were generated with the DiPer migration tool for Microsoft Excel. *** $p<0.001$, MSD of TC in the presence of Meso-CAFs versus in the absence of Meso-CAFs at endpoints, one-way ANOVA with Dunnett's multiple comparisons test.

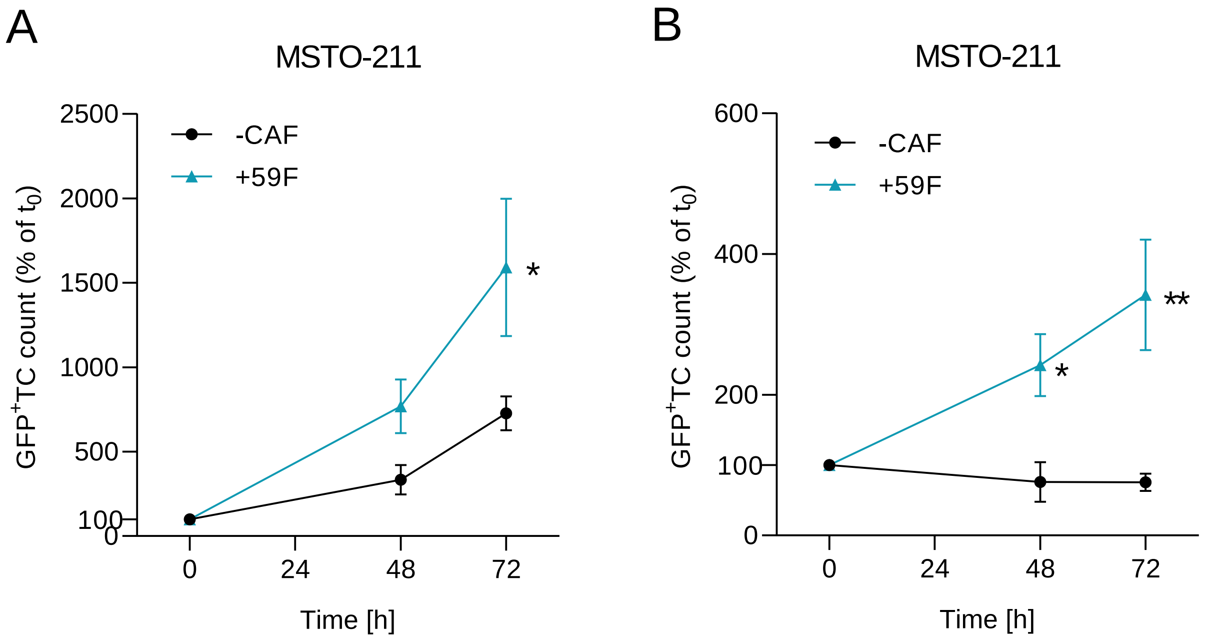

Supplementary Figure S12. Stimulation of MSTO-211H growth by VMC59F. Green fluorescent protein tagged (GFP^+) MSTO-211H were incubated in the presence and absence of VMC59 as 2D monolayers (A) or in 3D collagen gels (B). Micrographs were taken after 48 and 72 h and numbers of GFP^+ tumor cells (TC) were determined by automated image analysis. * $p<0.05$, ** $p<0.01$ TC number in the presence of VMC59F (+59F) versus TC number in the absence of VMC59F (-CAF), two-way ANOVA with Tukey's multiple comparisons test.

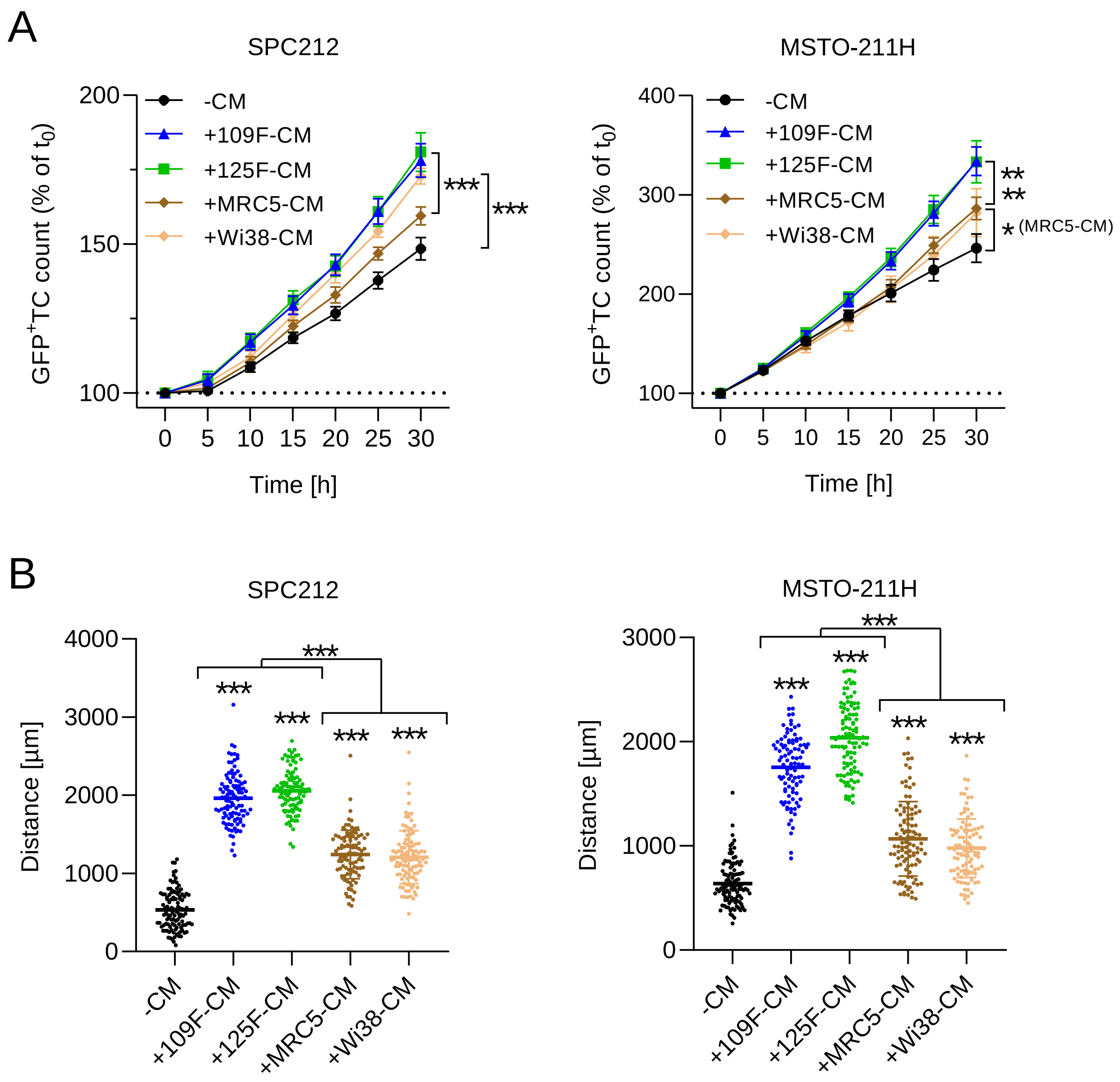

Supplementary Figure S13. Growth- and migration promoting effects of CM from Meso-CAFs versus NLFs. Green fluorescent protein tagged (GFP+) SPC212 cells (left panels) and MSTO-211H cells (right panels) were incubated without conditioned medium (-CM) or with CM conditioned by Meso109F (+109F-CM), Meso125F (+125F-CM), MRC-5 (+MRC5-CM) or Wi-38 (+Wi38-CM). (A) Micrographs were taken every 5 h and numbers of GFP+ tumor cells (TC) were determined by automated image analysis. (B) Micrographs were taken every 30 minutes and individual tumor cells (n > 90 per condition) from three biological replicates were manually tracked for 30 h. Each dot represents the migrated distance of one individual cell. . * $p<0.05$, ** $p<0.01$, *** $p<0.001$ TC number in the presence versus absence of CM or as indicated by brackets, two-way ANOVA with Tukey's multiple comparisons test (A) or one-way ANOVA with Dunnett's multiple comparisons test (B). Curves for growth stimulation by Meso-CAF-CM versus -CM are the same as shown in Figure 5 and statistical comparison between Meso-CAF-CM versus -CM is only shown there.

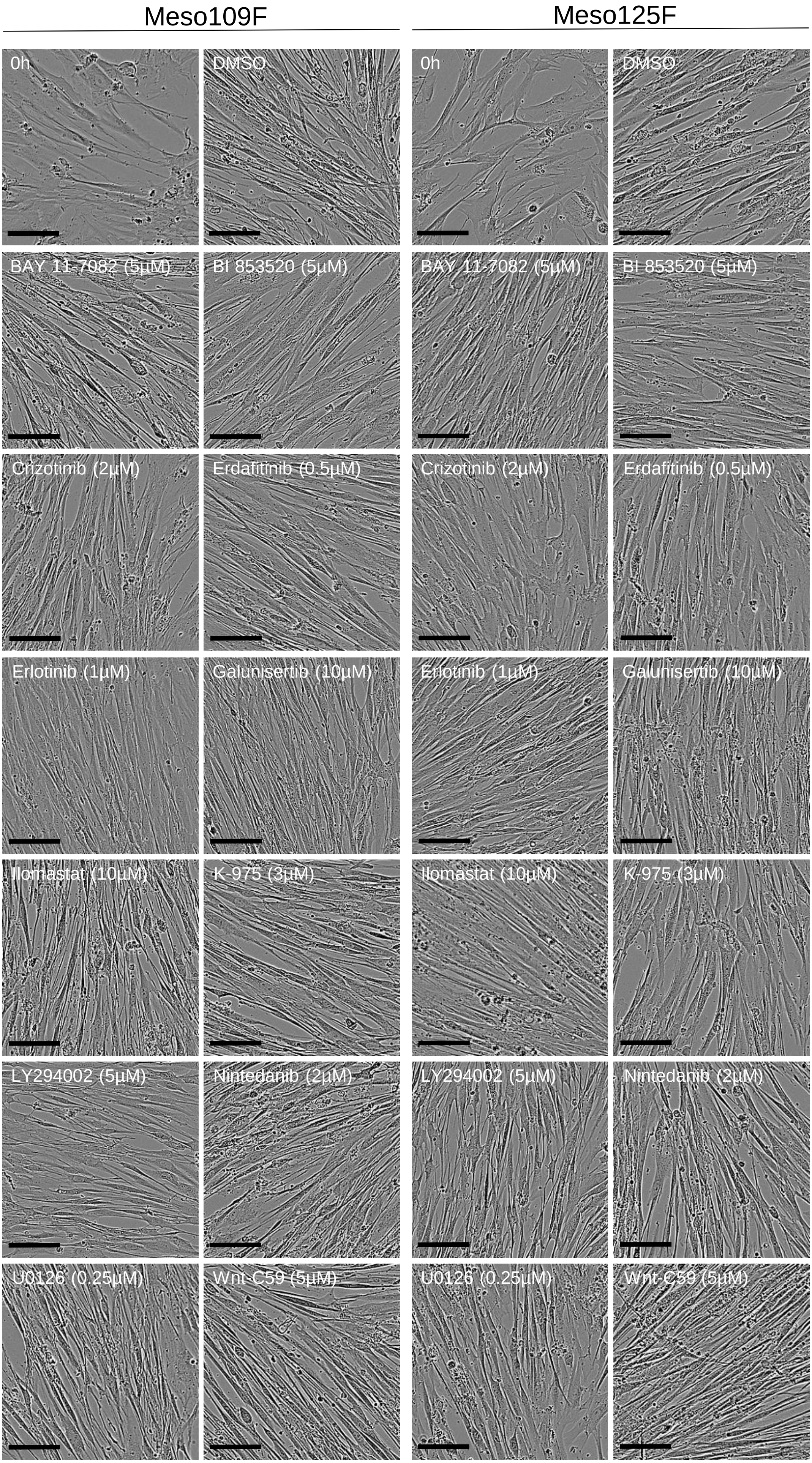

Meso109F
Meso125F
0h
DMSO
BAY 11-7082 (5μM)
BI 853520 (5μM)
Crizotinib (2μM)
Erdafitinib (0.5μM)
Erlotinib (1μM)
Galunisertib (10μM)
Ilomastat (10μM)
K-975 (3μM)
LY294002 (5μM)
Nintedanib (2μM)
U0126 (0.25μM)
Wnt-C59 (5μM)

Supplementary Figure S14. Absence of inhibitor-induced cytotoxicity in Meso-CAFs. Micrographs were taken after 72 h of inhibitor treatment at the indicated concentrations. DMSO was used as vehicle in all cases and is shown for comparison. Meso-CAFs before treatment start (0 h) are also shown. Scale bar = 100 µm.

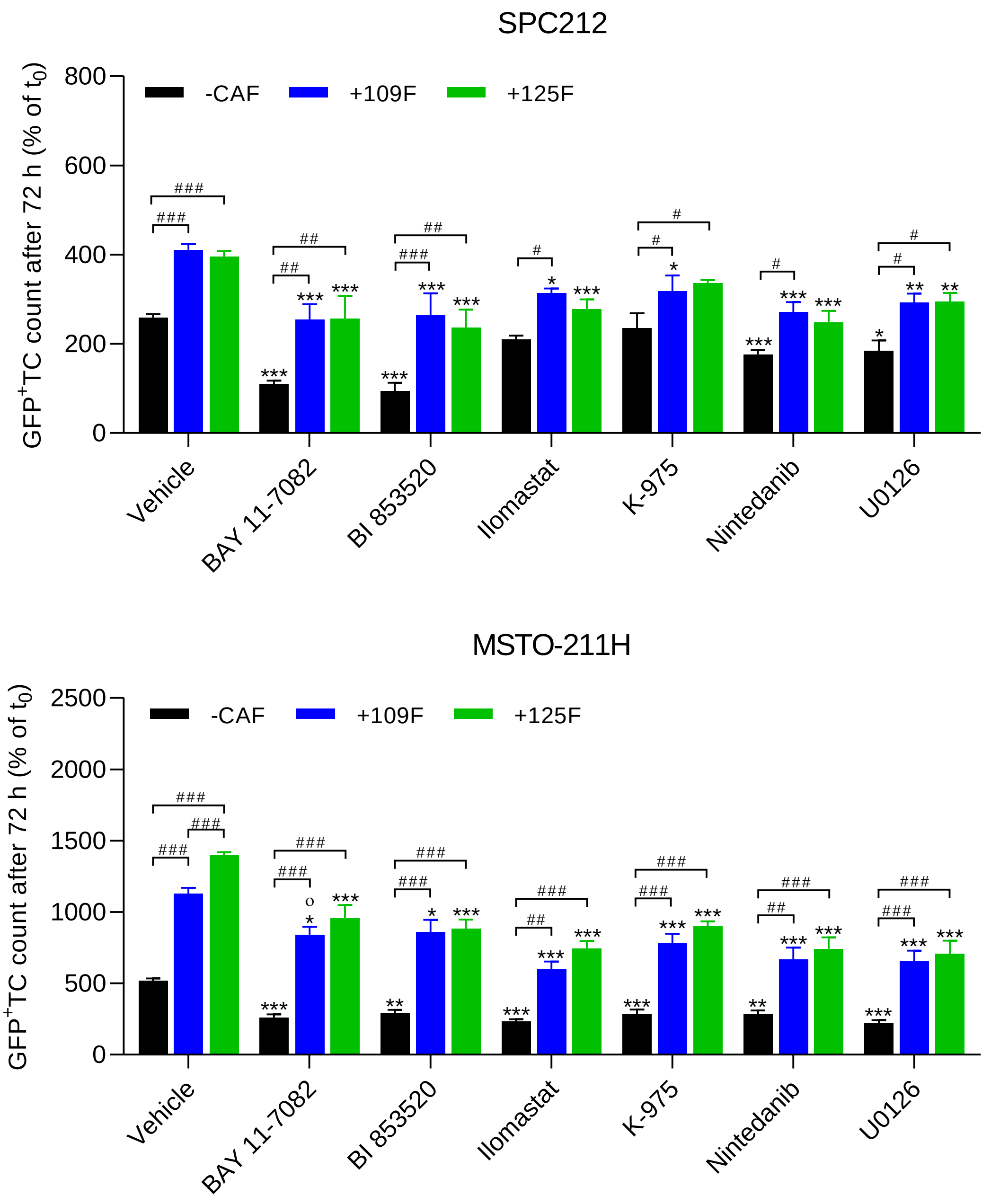

Supplementary Figure S15. Response of PM cells to signaling pathway inhibitors in the presence or absence of Meso-CAFs. GFP+ SPC212 (upper panel) or GFP+ MSTO-211H (lower panel) were cultured in the absence of CAFs (-CAF) or in the presence of Meso109F (+109F) or Meso125F (+125F) and treated with BAY 11-7082, BI 853520, ilomastat, K-975, nintedanib, U0126 or vehicle (DMSO). Numbers of GFP+ TC after 72 h were calculated from micrographs by automated image analysis. ** p<0.01, *** p<0.001 inhibitor treated versus vehicle treated, # p<0.05, ## p<0.01, ### p<0.001 growth in presence of Meso-CAFs versus growth in absence of Meso-CAFs, ° p<0.05, °° p<0.01, °°° p<0.001 percent inhibition in presence of Meso-CAFs versus percent inhibition in absence of Meso-CAFs, one-way ANOVA with Tukey's multiple comparisons test.

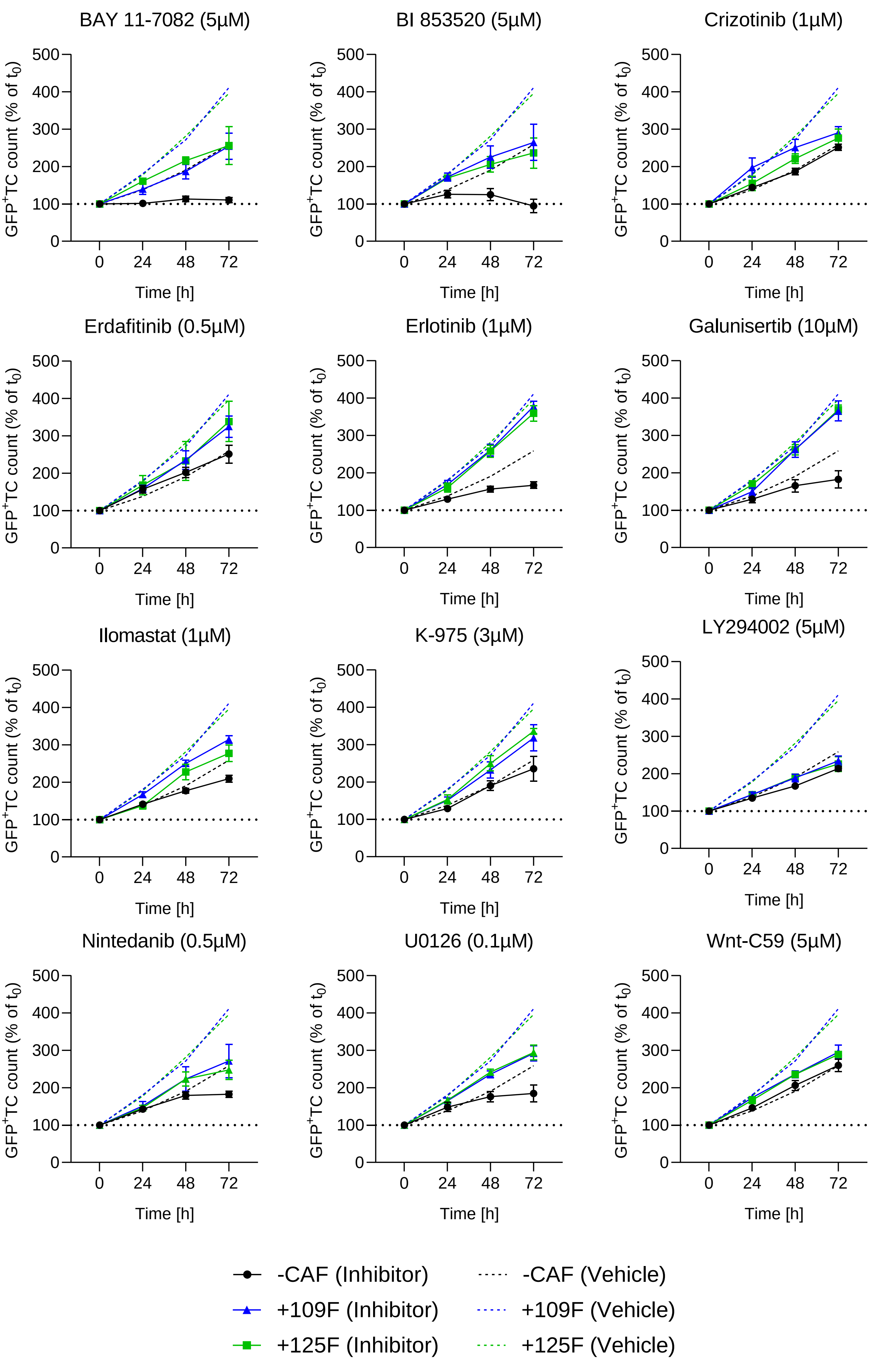

BAY 11-7082 (5µM)
BI 853520 (5µM)
Crizotinib (1µM)
Erdafitinib (0.5µM)
Erlotinib (1µM)
Galunisertib (10µM)
Ilomastat (1µM)
K-975 (3µM)
LY294002 (5µM)
Nintedanib (0.5µM)
U0126 (0.1µM)
Wnt-C59 (5µM)
GFP+TC count (% of t0)
Time [h]
-CAF (Inhibitor)
+109F (Inhibitor)
+125F (Inhibitor)
-CAF (Vehicle)
+109F (Vehicle)
+125F (Vehicle)

Supplementary Figure S16. Time course of SPC212 cell response to inhibitors in the presence or absence of Meso-CAFs. GFP^+ SPC212 were cultured in the absence of CAFs (-CAF) or in the presence of Meso109F (+109F) or Meso125F (+125F) and treated with the indicated inhibitors or vehicle (DMSO). Micrographs were taken every 24 h and numbers of GFP^+ tumor cells (TC) were calculated by automated image analysis. Statistical evaluation at endpoint (72 h) is shown in Figure 5 and Supplementary Figure 15.

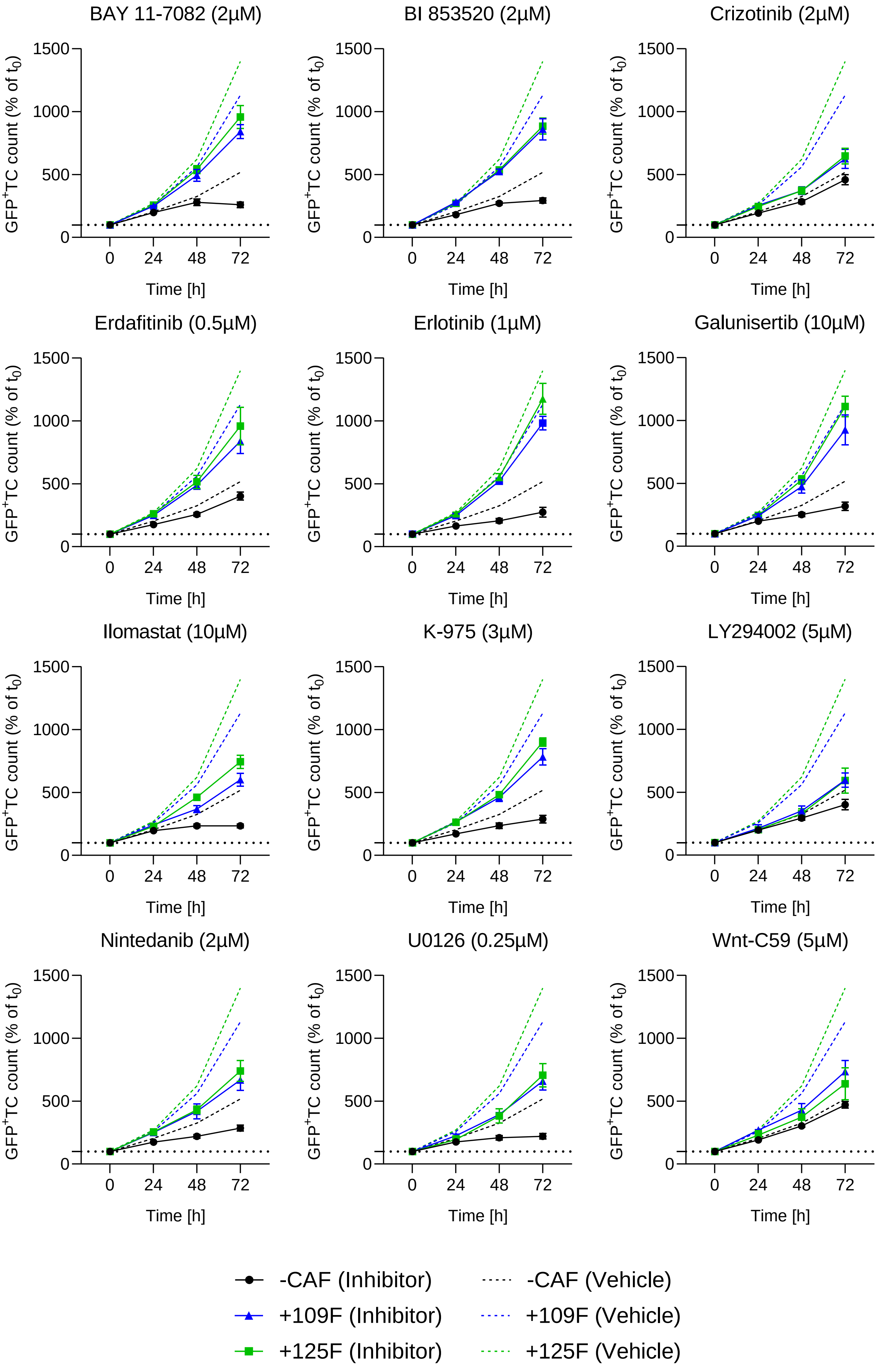

BAY 11-7082 (2µM)
BI 853520 (2µM)
Crizotinib (2µM)
Erdafitinib (0.5µM)
Erlotinib (1µM)
Galunisertib (10µM)
Ilomastat (10µM)
K-975 (3µM)
LY294002 (5µM)
Nintedanib (2µM)
U0126 (0.25µM)
Wnt-C59 (5µM)
GFP+TC count (% of t0)
Time [h]
-CAF (Inhibitor)
+109F (Inhibitor)
+125F (Inhibitor)
-CAF (Vehicle)
+109F (Vehicle)
+125F (Vehicle)

Supplementary Figure S17. Time course of MSTO-211H cell response to inhibitors in the presence or absence of Meso-CAFs. GFP$^+$ MSTO-211H were cultured in the absence of CAFs (-CAF) or in the presence of Meso109F (+109F) or Meso125F (+125F) and treated with the indicated inhibitors or vehicle (DMSO). Micrographs were taken every 24 h and numbers of GFP$^+$ tumor cells (TC) were calculated by automated image analysis. Statistical evaluation at endpoint (72 h) is shown in Figure 5 and Supplementary Figure 15.
